# Irisin regulates oxidative stress and mitochondrial dysfunction through the UCP2-AMPK pathway in prion diseases

**DOI:** 10.1038/s41419-025-07390-w

**Published:** 2025-02-03

**Authors:** Pei Wen, Zhixin Sun, Dongming Yang, Jie Li, Zhiping Li, Mengyang Zhao, DongDong Wang, Fengting Gou, Jingjing Wang, Yuexin Dai, Deming Zhao, Lifeng Yang

**Affiliations:** National Key Laboratory of Veterinary Public Health and Safety, Key Laboratory of Animal Epidemiology of the Ministry of Agriculture and Rural Affairs, National Animal Transmissible Spongiform Encephalopathy Laboratory, College of Veterinary Medicine, China Agricultural University, Beijing, China

**Keywords:** Apoptosis, Mechanisms of disease

## Abstract

Prion diseases are a group of fatal neurodegenerative disorders characterized by the abnormal folding of cellular prion proteins into pathogenic forms. The development of these diseases is intricately linked to oxidative stress and mitochondrial dysfunction. Irisin, an endogenous myokine, has demonstrated considerable neuroprotective potential due to its antioxidative properties. However, the protective effects of irisin against prion diseases have yet to be clarified. Our findings indicate that treatment with exogenous irisin can mitigate the apoptosis induced by PrP^106–126.^ Additionally, irisin significantly reduces oxidative stress and alleviates the mitochondrial dysfunction triggered by PrP^106–126^. Furthermore, irisin treatment targets uncoupling protein 2 (UCP2) and activates the AMPK-Nrf2 pathway, substantially improving oxidative stress and mitochondrial dysfunction in N2a cells induced by PrP^106–126^. These results suggest that irisin represents a novel and promising therapeutic approach for treating prion diseases.

## Highlights

- PrP^106–126^ induces mitochondrial dysfunction in a mtROS-dependent manner.
- Irisin alleviates PrP^106–126^-induced oxidative stress via UCP2 activation.
- UCP2 mediates irisin-induced AMPK-Nrf2 activation.
- Inhibition of oxidative stress rescues PrP^106–126^-induced cell death.

## Graphical abstract

**Figure:**
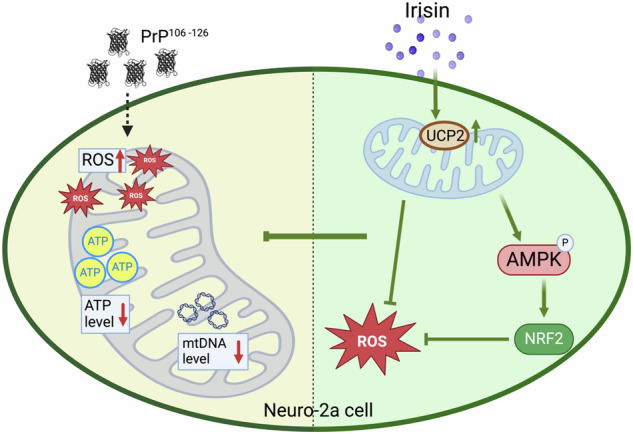
PrP^106–126^ exposure induces ROS accumulation, leading to mitochondrial dysfunction and cell death. Irisin has a protective effect against PrP^106–126^ toxicity and depends on the activation of the UCP2-AMPK signaling pathway.

## Introduction

Prion diseases constitute a group of fatal neurodegenerative disorders, that are distinctively marked by the aberrant folding of the cellular prion protein (PrP^C^) into its pathogenic isoform (PrP^Sc^) [1, 2]. The PrP^106-126^ peptide, representing a specific segment of the prion protein, has emerged as a pivotal tool in the investigation of these conditions [3, 4]. This synthetic peptide, which mirrors residues 106–126 of the human prion protein, successfully mimics several hallmark features of PrP^Sc^, such as cytotoxicity, neurotoxicity, gliotrophic activity, resistance to proteinase-K, and β-sheet conformation [5, 6]. PrP^106–126^ contributes to our understanding of the role that amyloid-like structures play in the neurodegenerative pathways of prion diseases, highlighting the critical role of oligomers and prion-like spread in protein misfolding disorders [7, 8]. Overall, the PrP^106–126^ peptide serves as a vital instrument in prion research, elucidating the molecular underpinnings of prion-induced neurotoxicity and facilitating the development of therapeutic approaches to combat these devastating diseases.

Mitochondria are essential organelles responsible for maintaining cellular physiological functions. However, they are highly vulnerable to oxidative stress, which can precipitate mitochondrial dysfunction and lead to subsequent cellular apoptosis [9]. Mitochondria are the primary source of cellular reactive oxygen species (ROS). Within the mitochondrial inner membrane, the uncoupling proteins (UCPs) family plays a critical role by partially reducing the proton gradient, thus regulating intracellular ROS homeostasis and mitigating oxidative stress [9]. UCP2, which is predominantly expressed in the nervous system, has been implicated in neurodegenerative diseases such as Parkinson’s disease (PD), Alzheimer’s disease (AD), and depression [10–12]. Its neuroprotective role is likely mediated by restricting ROS production and preserving mitochondrial function.

Additionally, AMPKα plays a crucial role in the regulation of oxidative stress and mitochondrial function. Its activation directly stimulates Nrf2, enhancing the expression of antioxidant genes, reducing Pb-induced ROS production, and preventing apoptosis [13]. Recent research has shown highlights that the phosphorylation of AMPK by UCP2 is essential [14]. Consequently, targeting UCP2 and AMPKα could have significant therapeutic relevance for addressing prion-induced oxidative stress and mitochondrial dysfunction. Although the significance of oxidative stress in the pathogenesis of prion diseases is well recognized, the detailed mechanisms involved still need further elucidation.

Recent discoveries have revealed that irisin, an endogenous myokine released into circulation through the cleavage of fibronectin type III domain-containing protein 5 (FNDC5), and it has garnered considerable attention for its anti-inflammatory and antioxidant properties [15–17]. Previous studies have demonstrated that irisin can cross the blood-brain barrier (BBB) and accumulate in the brain, thereby exerting neuroprotective effects [18]. Consequently, irisin may represent an innovative therapeutic agent for prion diseases, by acting as a crucial mediator of neuronal health. Nonetheless, the ability of irisin to mitigate oxidative stress and mitochondrial dysfunction associated with prion diseases remains to be clarified. Therefore, this study sought to evaluate the protective effects of irisin on oxidative stress and mitochondrial function in a prion disease model and to explore the underlying mechanisms involved.

Our findings indicate that oxidative stress induced by prion disease, results in mitochondrial dysfunction. Irisin addresses these challenges by activating the UCP2/AMPK pathway, thereby inhibiting oxidative stress and ameliorating mitochondrial dysfunction in the model. Overall, these results underscore the potential therapeutic value of irisin in prion diseases and other neurodegenerative conditions characterized by oxidative stress.

## Materials and methods

### Ethical statement

This study does not involve direct intervention by human or animal entities and therefore does not require ethical approval.

### Cell culture

The N2a mouse neuroblastoma cell line (ATCC#CCL-131) and the SH-SY5Y Human neuroblastoma cell line (ATCC#CRL-2266), both verified to be free of mycoplasma contamination, were acquired from the Cell Resource Center of the Chinese Academy of Medical Sciences/Peking Union Medical College. The N2a cells were cultured in Dulbecco’s modified Eagle’s medium (DMEM) supplemented with 10% fetal bovine serum, while the SH-SY5Y cells were cultured in Dulbecco’s modified Eagle’s medium/F12 (DMEM/F12) under the same supplementation. All cells were maintained at 37 °C in a humidified atmosphere with 5% CO_2._

The PrP^106–126^ peptide, provided by Amy Peptide Biotech, had a purity exceeding 98% and the sequence KTNMKHMAGAAAAGAVVGGLG. The scrambled sequence used was MEVGWYRSPFSRVVHLYRNGK. The peptide was dissolved in 0.1 M PBS to a storage concentration of 1 mM and agitated at 4 °C for 24 h to promote aggregation. The experiments were conducted under aseptic conditions, with a final peptide concentration of 150 µM used.

Irisin (HY-P70665, MCE, USA) was dissolved in DMEM and stored at −20 °C in a 1 mg/ml stock solution, with a final concentration of 100 ng/ml used in experiments. The AMPK activator AICAR (250 µM, HY-13417, MCE, USA) and AMPK inhibitor compound C (10 µM, HY-13418, MCE, USA) and proteasome inhibitor MG132 (10 µM, S2619, Selleck, USA) were added according to experimental groups.

### Plasmids and transfection

UCP2 siRNA (sense: 5′-CTAUGAAAUCTUUGGGCUUTT-3′; antisense: 5′-AAGCC CAAAGAUUUCAUAGTT-3′) and Nrf2 siRNA (sense: 5′-GAGUGGAGUGCCAGU CUTT-3′; antisense: 5′-AGACUGGCACUCACCACUCTT-3′), along with the pcDNA3.1(+)-UCP2 and pcDNA3.1(+)-Nrf2 plasmids, were acquired from Synbio Technologies. Mito-GFP and DsRed-Mito plasmids were purchased from Clontech. N2a cells were transfected using Lipofectamine 3000 (L3000015, Invitrogen, Carlsbad, CA, USA) in Opti-MEM (31985062, Gibco, California, USA).

### Cell viability assay

N2a cells were initially treated with irisin at concentrations of 10, 20, 50, or 100 ng/ml at 37 °C for 2 h, followed by the addition of 150 µM PrP^106–126^ and further incubation for 24 h. Cell viability was assessed using the Cell Counting Kit-8 (C0037, Beyotime, Shanghai, China). CCK-8 solution was added to the culture medium, and the samples were incubated at 37 °C with 5% CO_2_ for 1 h. The absorbance was measured at 450 nm using a microplate reader BioTek (Beijing, China), with background control samples used as the blank control group.

### Flow cytometry to detect cell apoptosis

To quantify the percentage of apoptotic cells, the cells were digested with 0.25% trypsin, and proteolysis was neutralized using 10% FBS. The cell suspensions were then centrifuged at 3000 rpm for 5 min, washed once with PBS, and stained with Annexin V-FITC and propidium iodide (PI) solution (C1062, Beyotime Biotechnology, Shanghai, China) for 15 min at room temperature in the dark. The percentage of apoptotic cells in each sample was subsequently analyzed using a BD FACSCalibur flow cytometer (BD Biosciences, Franklin Lakes, NJ, USA).

### TUNEL assay

Apoptosis in N2a cells was detected using a one-step TUNEL Apoptosis Assay Kit (C1086, Beyotime, Shanghai, China). Initially, N2a cells were seeded in 24-well plates and prepared according to the manufacturer’s instructions prior to peptide treatment. Subsequently, apoptosis was carefully observed and analyzed using an A1 confocal microscope (Nikon).

### Measurement of mitochondrial transmembrane potential and ATP levels

The mitochondrial membrane potential was assessed using the JC-1 Mitochondrial Membrane Potential Assay Kit (C2005, Beyotime, Shanghai, China). The cells were incubated in JC-1 staining solution at 37 °C with 5% CO_2_ for 20 min, and the samples were analyzed using flow cytometry.

ATP levels were measured using an ATP Assay Kit (S0026, Beyotime, Shanghai, China) with a microplate reader from BioTek (Beijing, China). All procedures were carried out following the manufacturer’s instructions.

### DNA isolation and mtDNA copy number assay

Total DNA was isolated from N2a cells using a Universal Genomic DNA Extraction Kit (CW2298S, CWBIO, Beijing, China), and quantified using spectrophotometry (NanoDrop 2000). The ViiA 7 Fast Real-Time PCR System (ABI) and SYBR Green Master Mix (Q141-02; Vazyme, Nanjing, China) were used for qPCR. Quantification was based on mtDNA and genomic DNA (gDNA), using the comparative CT method (2^−ΔΔCT^), and the primers used are listed in Table S1.

### Measurement of mitochondrial respiratory chain complexes

The activities of mitochondrial respiratory chain complexes I, II, III, and IV were assessed according to the manufacturer’s instructions (Solarbio, Beijing, China). The assays were performed using a microplate reader from BioTek (Beijing, China).

### Oxidative stress measurement

Antioxidant defense markers in N2a cells, including superoxide dismutase (SOD), catalase (CAT), and malondialdehyde (MDA), and the glutathione/oxidized glutathione ratio (GSH/GSSG), in N2a cells were assessed following the manufacturer’s instructions (Nanjing Jiancheng Bioengineering Institute, China). The assays were conducted using a microplate reader from BioTek (Beijing, China).

### Flow cytometry to detect mtROS

To measure the mtROS content in the cells, the cells were digested with 0.25% trypsin and then neutralized with 10% fetal bovine serum to neutralize proteolysis. The cell suspension was then centrifuged at 3000 rpm for 5 min, and washed once with PBS, and the cells were incubated with MitoSOX (2 µM, Thermo Fisher Scientific, Sunnyvale, CA, USA) for 30 min, followed by two PBS washes and flow cytometric analysis (BD Biosciences, Franklin Lakes, NJ, USA).

### Immunofluorescence staining

Prior to treatment, mitochondrial N2a cells were transfected with DsRed-Mito or siRNA for 48 h, followed by treatment with PrP^106–126^, Mito-tempo, FTIC-irisin (MCE, USA), and irisin. The cells were washed twice with PBS and fixed with 4% paraformaldehyde for 30 min. The cells were then permeabilized with immunostaining permeabilization buffer containing Triton X-100 (Beyotime Biotechnology, P0096) at room temperature for 5 min. After blocking for 1 h at room temperature, the cells were incubated overnight at 4°C with specific primary antibodies (as described in the immunoblotting section). Secondary antibodies were applied for 1 h at 37 °C, followed by washing and mounting with an anti-fade reagent containing DAPI. Fluorescence images were acquired using an A1 confocal microscope (Nikon, Tokyo) and quantified using ImageJ software.

### Transmission electron microscopy (TEM)

For TEM morphological analysis, N2a cells were collected and centrifuged in centrifuge tubes, followed by fixation with 2.5% glutaraldehyde at 4 °C for 12 h. The samples were then embedded and observed using a transmission electron microscope (HITACHI HT7700, Japan).

### Western blot

The N2a cells were homogenized using RIPA buffer (R0010, Solarbio Life Sciences, Beijing, China). After homogenization, the lysates were incubated on ice for 30 min and centrifuged at 12,000 rpm for 10 min at 4 °C. Nuclear and cytoplasmic proteins were extracted using the Nuclear and Cytoplasmic Protein Extraction Kit (Beyotime, P0028) according to the manufacturer’s instructions. Mitochondrial and cytosolic proteins were extracted using the Cell Mitochondria Isolation Kit (Beyotime, C3601) according to the manufacturer’s instructions. Protein concentrations were determined using the BCA method. Equal amounts of protein were resolved on 10–15% SDS-PAGE gels and then transferred onto PVDF membranes using a standard protocol. This membrane was blocked with 5% skim milk in TBST for 90 min at room temperature. It was then incubated overnight at 4 °C with primary antibodies (see Table S2), followed by a 2-h incubation at room temperature with HRP-conjugated goat anti-mouse IgG or goat anti-rabbit IgG (see Table S2). The blots were visualized using a Chemiluminescent Imaging System (Tanon Science and Technology).

### Real-time quantitative PCR

N2a cell samples obtained from experiments were first homogenized using TRIzol reagent (R401-01, Vazyme, Nanjing, China). Following the manufacturer’s guidelines, total RNA was extracted from the homogenized tissue. After determining the concentration of the RNA samples, 1 µg of RNA was reverse transcribed using HiScript II Select SuperMix (R312-02, Vazyme, Nanjing, China) to generate cDNA. The results were was analyzed using real-time quantitative PCR (qPCR), which was performed using gene-specific primer sets and SYBR Green Master Mix (Q141-02; Vazyme, Nanjing, China). Changes in fluorescence changes were monitored using the ViiA 7 Fast Real-Time PCR System (ABI) to record and analyze the experimental process. The primers utilized are listed in Table S1.

### Statistical analysis

The data are presented as the mean ± standard deviation (SD) and were analyzed using Prism 8.0 software. Student’s *t*-test was used to compare differences between two groups. Differences between multiple groups were compared by a one-way ANOVA followed by Tukey’s post hoc test. Significance was considered at a level of *p* < 0.05.

## Results

### Irisin reduces PrP^106–126^-induced neuronal apoptosis

Previous research from our group indicated that cell death in N2a cells induced by the PrP^106–126^ peptide might be mediated by the activation of the mitochondrial-dependent apoptosis pathway [19]. To assess the effect of irisin on this process, we performed in vitro supplementation experiments with irisin in the PrP^106–126^-induced N2a cell model. Initially, the CCK-8 assay results demonstrated that the viability of N2a cells significantly decreased after treatment with 150 µM PrP^106–126^. We observed that irisin, at concentrations of 100 ng/mL and above, irisin effectively mitigated the dose-dependent detrimental effects of PrP^106–126^ on cell viability (Fig. S1A, B). Based on these findings, we selected 150 µM PrP^106–126^ and 100 ng/mL irisin for treatment in subsequent studies.

**Fig. 1:**
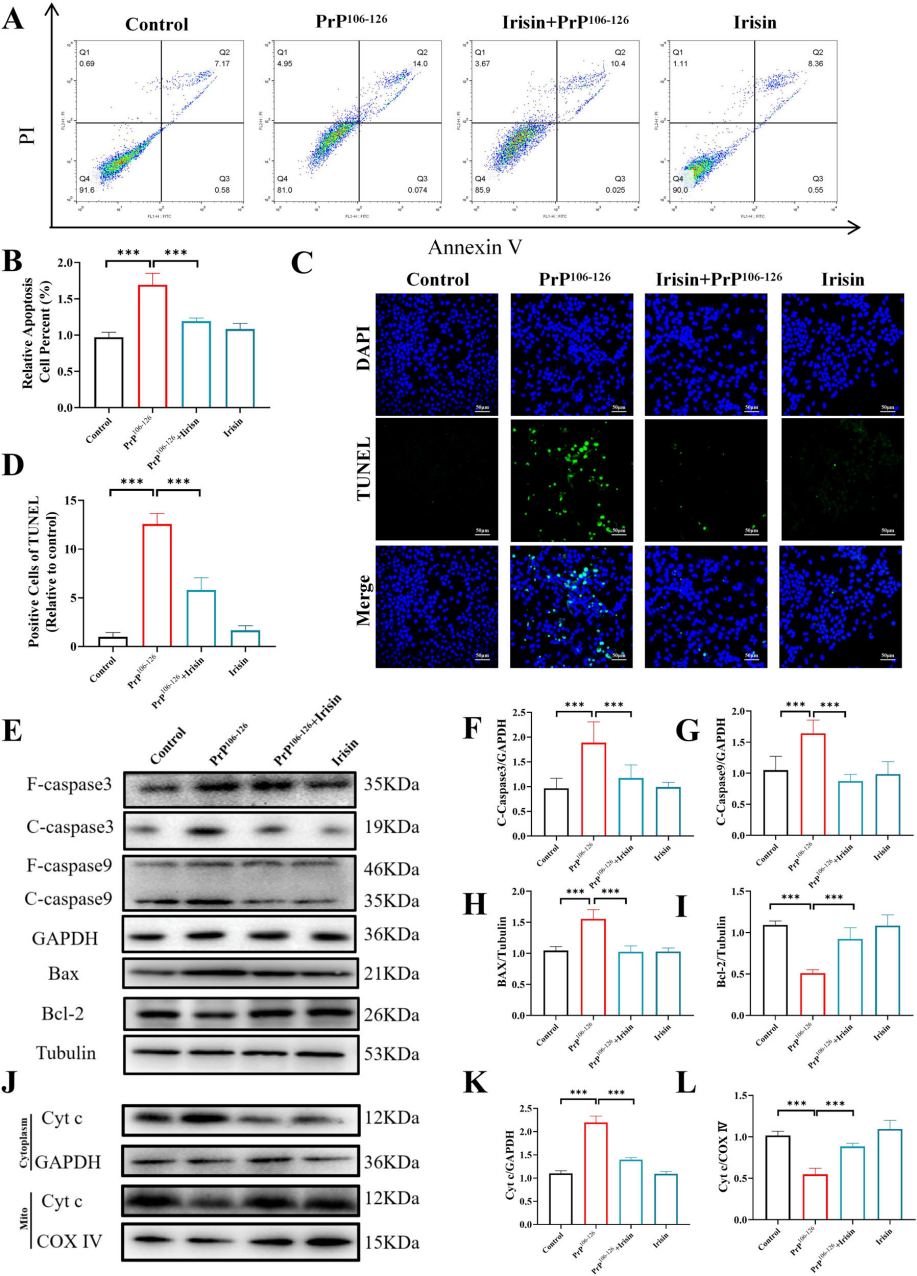
Irisin reduces PrP^106–126^-induced neuronal apoptosis. **A**, **B** After N2a cells were treated with irisin and PrP^106–126^, apoptosis was assessed by Annexin V-FITC/PI flow cytometry, and cell apoptosis was measured. **C**, **D** Determination of N2a cell apoptosis by TUNEL staining, (scale bar = 50 µm). **E**–**I** Detection of cleaved caspase-3, cleaved caspase-9, Bax and Bcl2 protein expression in N2a cells by western blotting. **J**–**L** The cytochrome c protein expression in cytosolic and mitochondrial extracts in N2a cells. The data are presented as the means ± SDs (*n* = 6), *P < 0.05; **P < 0.01; ***P < 0.001.

Apoptotic responses were evaluated using Annexin V binding assays with fluorescence labeling, which revealed a significant increase in apoptosis in N2a cells treated with PrP^106–126^ compared to control cells. However, this increase was notably reduced in cells treated with both PrP^106–126^ and irisin (Fig. 1A, B). Similarly, TUNEL staining demonstrated that compared with PrP^106–126^ pretreatment alone, irisin pretreatment significantly decreased the number of apoptotic cells (Fig. 1C, D). Additionally, Western blot analyses indicated that irisin treatment substantially diminished the activation of caspase-3 and caspase-9, decreased the Bax/Bcl-2 ratio, and reduced cytochrome c release in N2a cells exposed to PrP^106–126^ (Fig. 1E–L). Notably, similar results were also observed in SH-SY5Y cells (Fig. S1C–F), further supporting the notion that irisin effectively suppresses the mitochondria-dependent apoptotic pathway.

### Irisin alleviates PrP^106–126^-induced neuronal mitochondrial dysfunction in N2a cells

**Fig. 2:**
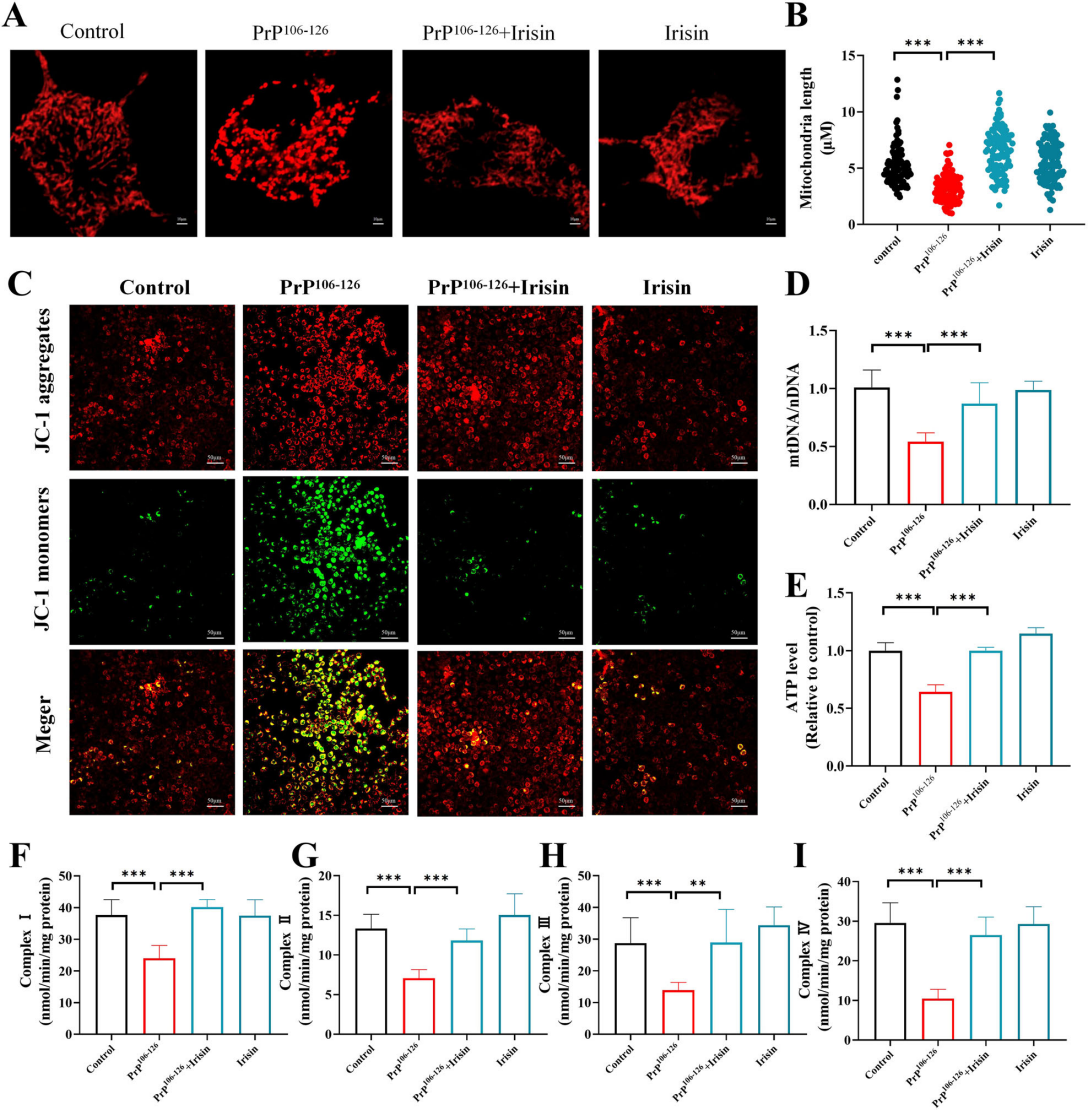
Irisin alleviates PrP^106–126^-induced neuronal mitochondrial dysfunction in N2a cells. **A**, **B** Mitochondrial morphology was measured by confocal microscopy, and mitochondrial length was analyzed by ImageJ software (scale bar = 10 µm). **C** Mitochondrial membrane potential (MMP) measurement by JC-1 dye in N2a cells (scale bar = 50 µm). **D** The mtDNA/nDNA ratio was assessed by real-time PCR. **E** Measurement of ATP levels in N2a cells. **F**–**I** The activity of the mitochondrial respiratory chain complexes (I–IV). The data are presented as the means ± SDs (*n* = 6), *P < 0.05; **P < 0.01; ***P < 0.001.

Mitochondrial dysfunction acts as a pivotal mediator of cellular apoptosis and is a crucial factor contributing to cell death [20, 21]. In our evaluation of the protective effects of irisin against neurotoxicity mediated by PrP^106–126^, we explored the underlying mechanisms by which irisin reduces mitochondrial dysfunction. In the control group, N2a cells displayed an intact mitochondrial network (Fig. 2A, B). However, in the PrP^106–126^-treated group, there was notable fragmentation of this network, indicating mitochondrial structural damage. In contrast, cells treated with irisin showed improved mitochondrial network integrity (Fig. 2A, B). The integrity of the mitochondrial network is essential for its function and ultimately determines its cellular fate. The reduction in the mitochondrial membrane potential observed in the PrP^106–126^ treatment group that highlighted mitochondrial dysfunction was ameliorated following irisin administration (Figs. 2C and S2A–C). Additionally, irisin pretreatment improved the reduction in mitochondrial DNA (mtDNA) copy number induced by PrP^106–126^, indicating that irisin can prevent the mitochondrial damage initiated by PrP^106–126^ (Fig. 2D).

Moreover, ATP, a key indicator of cellular energy metabolism, was significantly depleted in the PrP^106–126^-treated group but was substantially elevated in the irisin-treated group (Fig. 2E). This finding suggested that irisin may facilitate the recovery of mitochondrial function by enhancing energy metabolism. The alterations in mitochondrial membrane potential and ATP levels coincided with changes in the activities of the mitochondrial respiratory chain complexes (complexes I–IV). Treatment with PrP^106–126^ markedly reduced the activity of these complexes, whereas treatment with irisin significantly restored their activity (Fig. 2F–I). Overall, irisin effectively reverses the mitochondrial dysfunction induced by PrP^106–126^, highlighting its potential as a therapeutic agent for treating mitochondria-related diseases.

### Irisin alleviates PrP^106–126^-induced mitochondrial dysfunction by mitigating oxidative stress in N2a cells

Recognizing oxidative stress as a critical driver of mitochondrial dysfunction [22, 23], we aimed to examine its role in the mitochondrial impairments observed with PrP^106–126^ exposure. We used Mito-Tempo, a targeted superoxide dismutase mimic, to counteract mitochondrial dysfunction. The experimental findings indicated that N2a cells treated with PrP^106–126^ displayed elevated mtROS levels, and Mito-Tempo treatment significantly reduced mtROS levels (Fig. S3A, B). Additionally, Mito-Tempo treatment significantly decreased malondialdehyde (MDA) levels (Fig. S3C), increased the reduced glutathione (GSH)-to-oxidized glutathione (GSSG) ratio (Fig. S3D), and enhanced the activities of catalase (CAT) and total superoxide dismutase (T-SOD) (Figs. S3E, F). Mito-Tempo also effectively restored the structural integrity and functional capacity of the mitochondrial network in N2a cells (Figs. S3G, H and S4A–F). These results suggest that PrP^106–126^-induced oxidative stress can lead to mitochondrial dysfunction, and that the antioxidant Mito-Tempo can effectively prevent this damage, thereby protecting mitochondrial function.

**Fig. 3:**
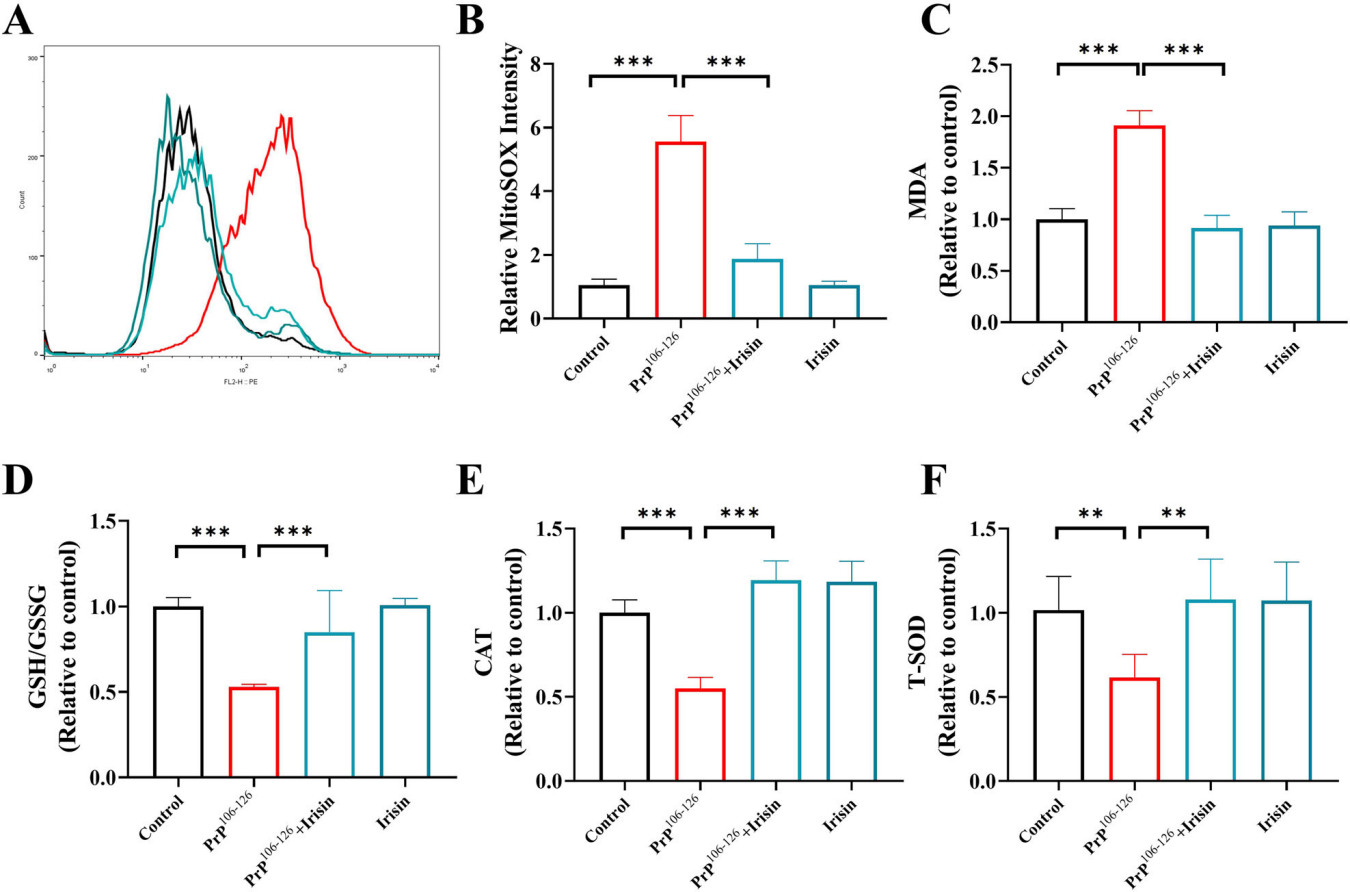
PrP^106–126^-induced oxidative stress leads to mitochondrial dysfunction in N2a cells. **A**, **B** Detection of mtROS production in N2a cells treated with irisin and PrP^106–126^, analyzed using MitoSOX staining and flow cytometry. **C**–**F** Measurement of MDA content, GSH/GSSH ratio, and CAT and T-SOD activities in N2a cells. The data are presented as the means ± SDs (*n* = 6), *P < 0.05; **P < 0.01; ***P < 0.001.

Furthermore, the antioxidant effects of irisin were assessed given its reported properties. Irisin treatment significantly decreased mtROS levels in PrP^106–126^-treated N2a cells (Fig. 3A, B). Compared with PrP^106-126^ treatment alone, irisin treatment notably reduced cellular MDA levels (Fig. 3C), improved the GSH/GSSG ratio (Fig. 3D), and increased the activities of CAT and T-SOD (Fig. 3E, F). These findings consistently demonstrate that irisin can alleviate the mitochondrial dysfunction induced by PrP^106–126^, and that this effect may be achieved by protecting against oxidative stress.

### UCP2 was responsible for the irisin-mediated beneficial effect

**Fig. 4:**
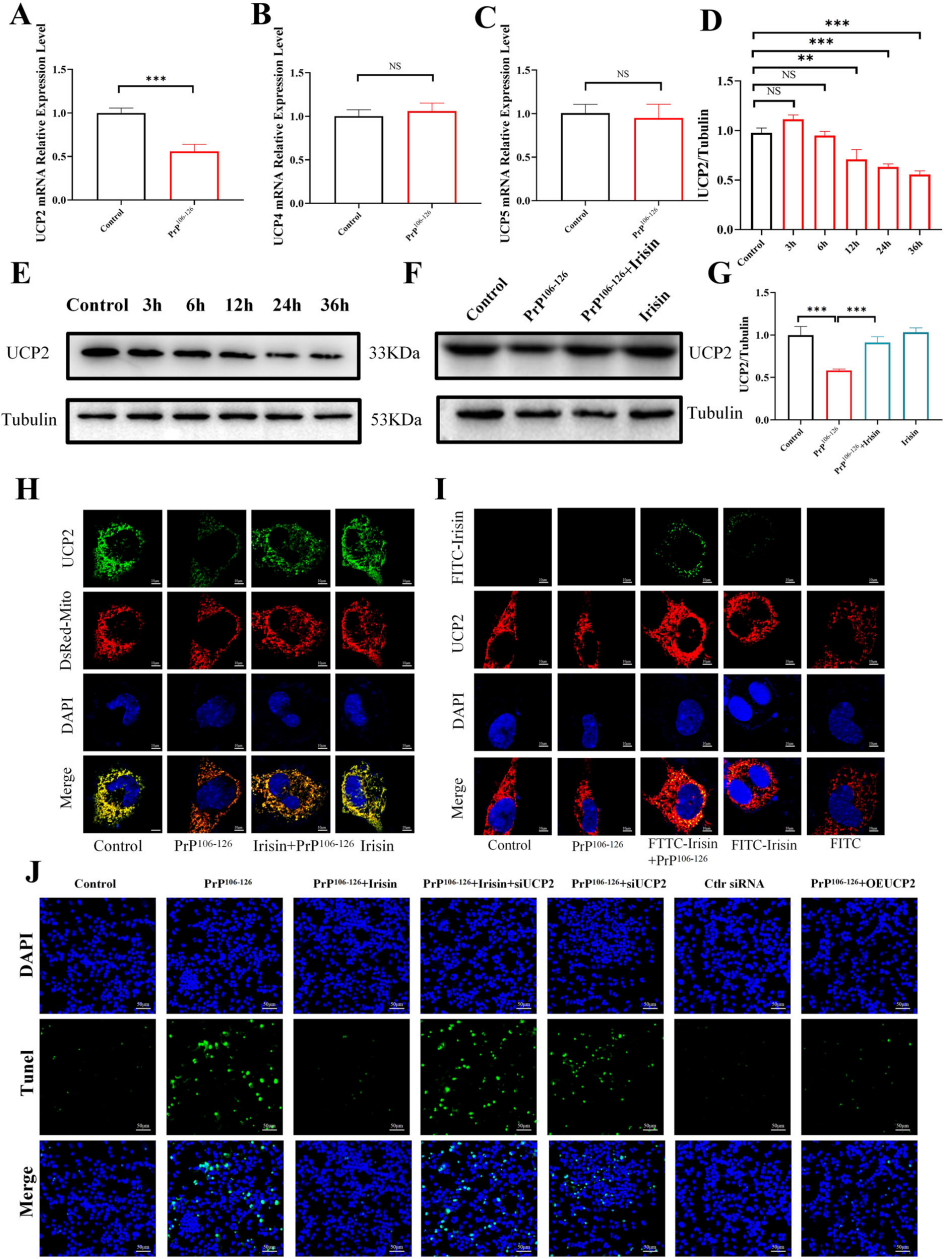
UCP2 is responsible for the Irisin-mediated beneficial effect in N2a cells. **A**–**C** The mRNA levels of UCP2, UCP4, and UCP5 in N2a cells after prp^106–126^ treatment, t test; **D**, **E** changes in UCP2 protein expression in N2a cells after prp^106–126^ treatment. **F**, **G** The protein expression level of UCP2 in N2a cells treated with irisin and PrP^106–126.^ **H** Immunofluorescence colocalization detection of the mitochondrial markers DsRed-Mito and UCP2 in N2a cells treated with irisin and PrP^106–126^ (scale bar = 10 µm). **I** Immunofluorescence colocalization of FITC-irisin and UCP2 in N2a cells treated with irisin and PrP^106–126^ (scale bar = 10 µm). **J** Determination of N2a cell apoptosis by TUNEL staining (scale bar = 50 µm). The data are presented as the means ± SDs (*n* = 6), *P < 0.05; **P < 0.01; ***P < 0.001.

Previous research has demonstrated that irisin protects lung tissue from oxidative damage during ischemia‒reperfusion (I/R) by enhancing the expression of UCP2, a member of the uncoupling protein (UCP) family predominantly expressed in brain tissue [24]. Our results revealed that treatment with PrP^106–126^ significantly decreased the transcription levels of UCP2 in N2a cells, with no notable changes observed in the expression of UCP4 and UCP5 (Fig. 4A–C). Additionally, UCP2 protein expression decreased as a function of the duration of PrP^106–126^ treatment (Fig. 4D, E). In contrast, irisin treatment mitigated this downregulation of UCP2 induced by PrP^106–126^ (Fig. 4F, G). Notably, irisin enhanced the colocalization of UCP2 with mitochondria (Figs. 4H and S5A), suggesting that irisin may preserve mitochondrial function via UCP2. To investigate the potential mechanisms by which irisin exerts its protective effects through UCP2 after PrP^106-126^ treatment, we administered FITC-labeled irisin to N2a cells posttreatment and observed colocalization of FITC-irisin with UCP2 (Figs. 4I and S5B). To further substantiate the role of UCP2 in the protective effect of irisin on PrP^106–126^ treatment, we manipulated UCP2 expression in N2a cells via siRNA silencing or overexpression (Fig. S5C). We also found that knockdown of UCP2 did not affect the expression of UCP4 and UCP5 (Fig. S5D). The results demonstrated that UCP2 overexpression mitigated the apoptosis induced by PrP^106–126^, whereas the apoptosis-protective effect of irisin was abolished in UCP2-silenced N2a cells (Figs. 4J and S5E), indicating that UCP2 is crucial for the protective effect of irisin on PrP^106–126^-treated N2a cells.

### Irisin relieves oxidative stress and maintains mitochondrial function via UCP2

**Fig. 5:**
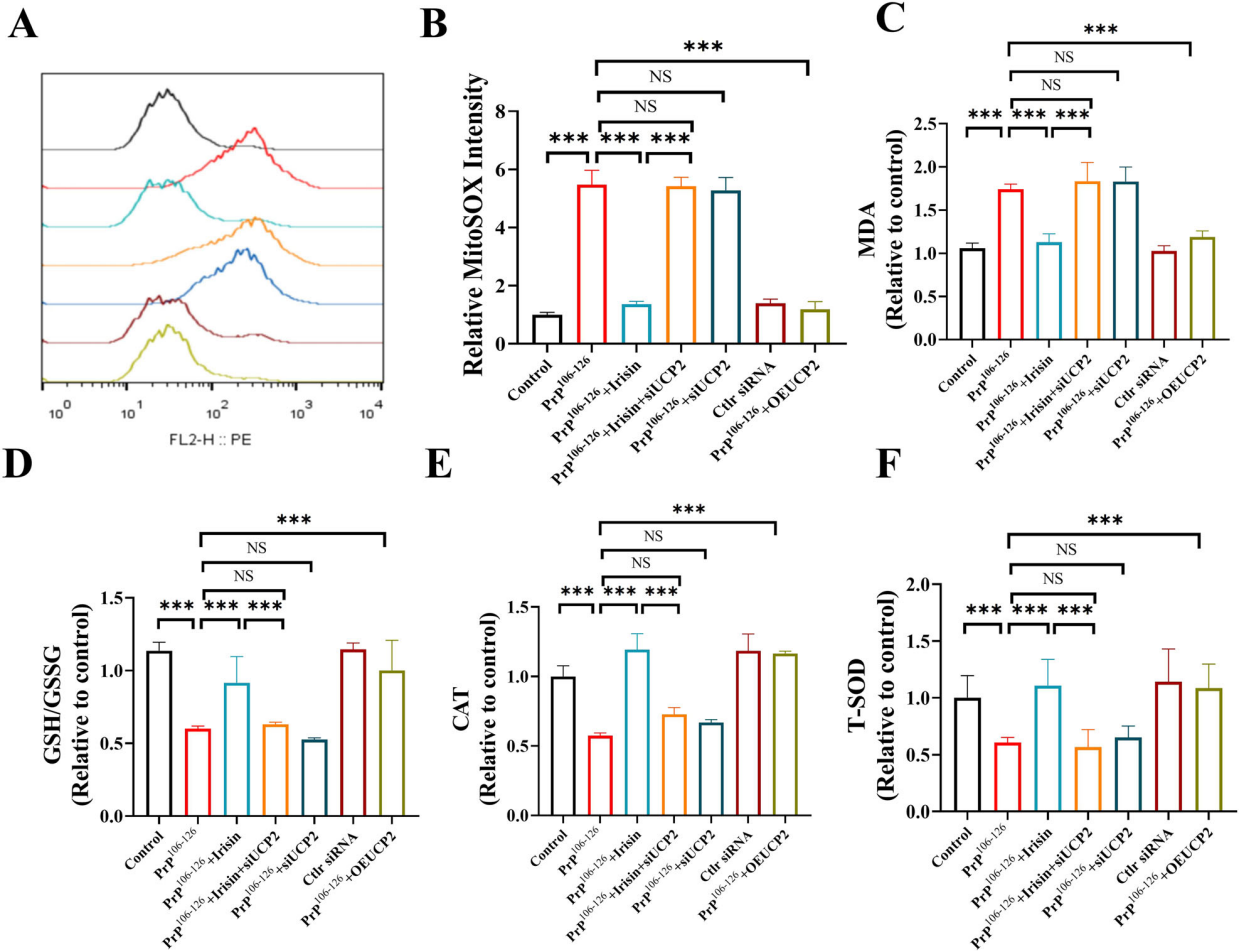
Irisin relieves oxidative stress via UCP2. **A**, **B** Detection of mtROS production in N2a cells after treatment, performed using MitoSOX staining and flow cytometry analysis. **C**–**F** Measurement of MDA content, GSH/GSSH ratio, and CAT and T-SOD activities in N2a cells. The data are presented as the means ± SDs (*n* = 6), *P < 0.05; **P < 0.01; ***P < 0.001.

**Fig. 6:**
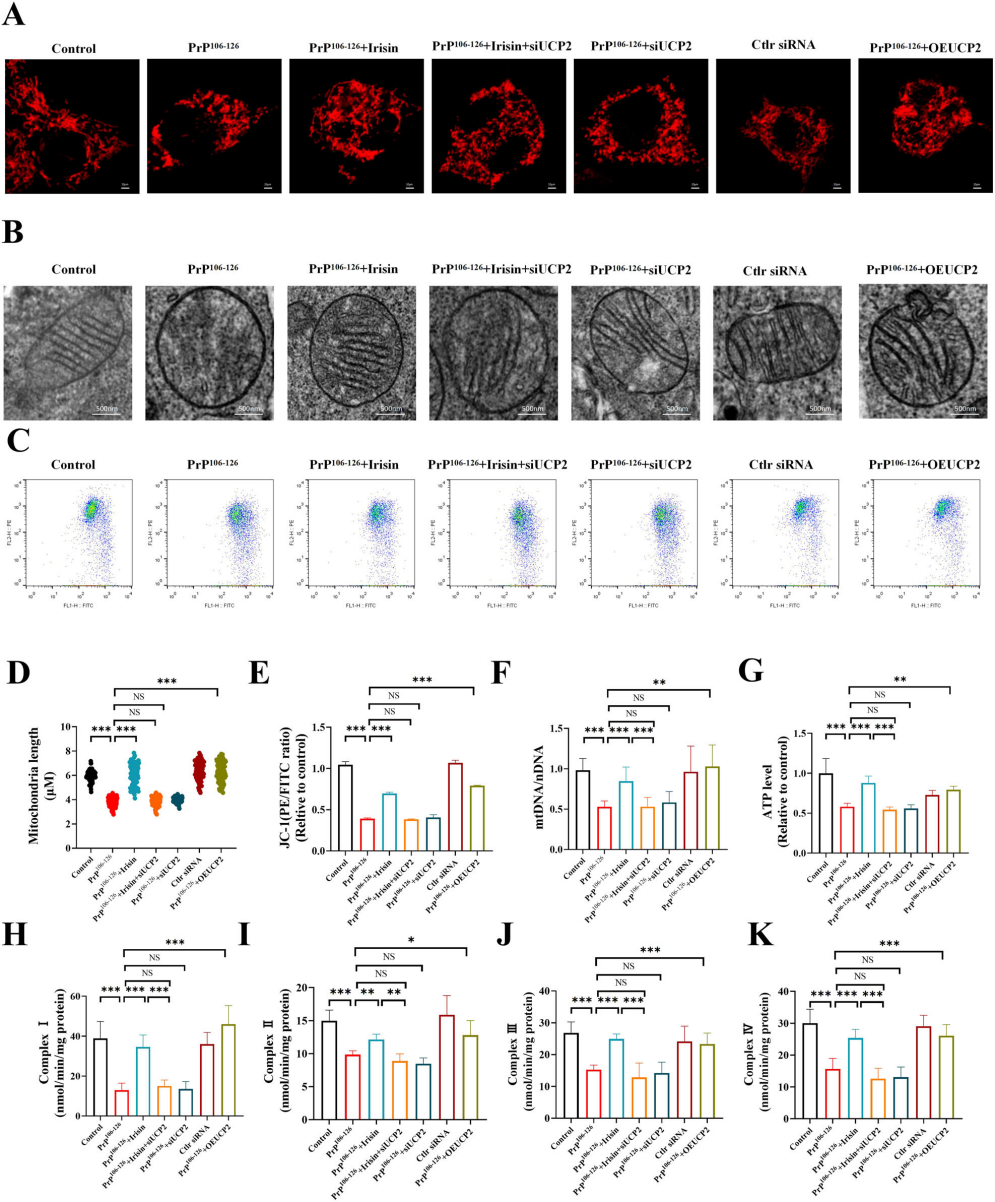
Irisin maintains mitochondrial function via UCP2. **A**, **D** The representative images of mitochondrial morphology and the quantification of mitochondrial length (scale bar = 10 µm). **B** Transmission electron microscopy was used to observe the mitochondrial ultrastructure of the cells (scale bar = 500 nm). **C**, **E** Measurement of mitochondrial membrane potential by JC-1 probes. **F** The mtDNA/nDNA ratio was assessed by real-time PCR. **G** Measurement of ATP levels. **H**–**K** The activity of the mitochondrial respiratory chain complexes (I–IV). The data are presented as the means ± SDs (*n* = 6), *P < 0.05; **P < 0.01; ***P < 0.001.

To elucidate the role of UCP2 in the antioxidant and mitochondrial protective effects mediated by irisin, we evaluated mitochondrial function in UCP2-knockdown N2a cells. Our findings revealed that the inhibitory effect of irisin on mtROS production was diminished upon UCP2 knockdown (Fig. 5A, B). Furthermore, the knockdown of UCP2 negated the protective effects of irisin against lipid peroxidation and oxidative damage in PrP^106–126^-treated N2a cells, as evidenced by unchanged levels of MDA (Fig. 5C), the GSH/GSSH ratio (Fig. 5D), CAT activity (Fig. 5E), and T-SOD levels (Fig. 5F). Additionally, UCP2 knockdown abolished the ability of irisin to protect mitochondrial function against PrP^106–126^-induced damage. In cells with reduced UCP2 expression, irisin was unable to mitigate the mitochondrial fragmentation induced by PrP^106–126^ (Fig. 6A, D). Electron microscopy further demonstrated that in UCP2-knockdown cells, irisin did not improve abnormalities in mitochondrial ultrastructure, such as diminished crista and swelling (Fig. 6B and Fig. S6A). Conversely, cells overexpressing UCP2 displayed improved mitochondrial cristae structure and reduced swelling (Fig. 6B). Moreover, irisin treatment failed to restore the mitochondrial membrane potential, ATP levels, mtDNA copy numbers, or the activities of electron transport chain complexes (I-IV) in UCP2-knockdown cells (Figs. 6E–K and S6B, C). These results substantiate the crucial role of UCP2 in facilitating the alleviation of oxidative stress and mitochondrial dysfunction induced by PrP^106–126^ by irisin.

### Irisin attenuates PrP^106–126^-induced oxidative stress via the UCP2-Nrf2 pathway in N2a cells

**Fig. 7:**
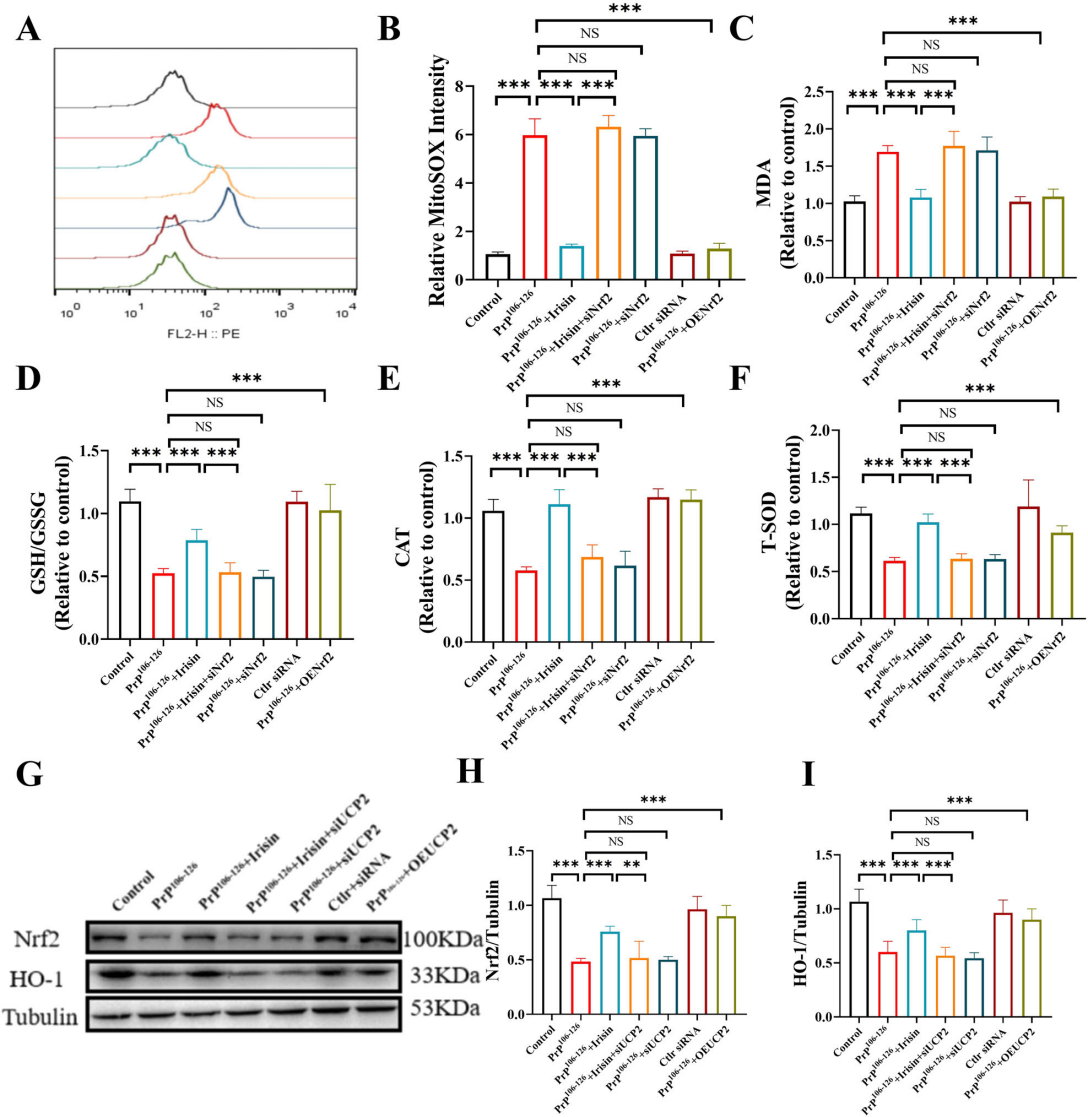
Irisin attenuates PrP^106–126^-induced oxidative stress via the UCP2-Nrf2 pathway in N2a cells. **A**, **B** Detection of mtROS production in N2a cells after treatment by flow cytometry analysis. **C**–**F** Measurement of MDA content, GSH/GSSH ratio, and CAT and T-SOD activities in N2a cells. **G**–**I** The protein expression levels of Nrf2 and HO-1 in N2a cells. The data are presented as the means ± SDs (*n* = 6), *P < 0.05; **P < 0.01; ***P < 0.001.

UCP2 mitigates oxidative stress by reducing mitochondrial ROS production [25]. Nrf2 is a pivotal regulator of the cellular defense system, facilitating the expression of various antioxidant and cytoprotective genes, including HO-1 and other detoxification enzymes [26]. The activation of these genes decreases mitochondrial ROS levels and reduces oxidative stress. In this study, we explored whether irisin and its downstream target, UCP2, counteract oxidative stress induced by PrP^106–126^ in N2a cells through the modulation of Nrf2 expression. Our findings showed that, compared to the control treatment, PrP^106–126^ treatment decreased Nrf2 and HO-1 levels, whereas irisin administration upregulated their expression (Fig. S7A, B). Consistent results were observed in SH-SY5Y cells, where PrP^106–126^ treatment also led to reduced Nrf2 and HO-1 levels, and irisin treatment successfully upregulated their expression (Fig. S7C, D). This finding suggested that irisin may confer antioxidant protection by enhancing the Nrf2 signaling pathway. To determine the role of Nrf2 in the antioxidant effects of irisin, we altered Nrf2 expression in N2a cells via knockdown and overexpression (Fig. S7E). The results indicated that overexpression of Nrf2 conferred resistance against oxidative damage triggered by PrP^106–126^ (Fig. 7A–F). In contrast, Nrf2 knockdown significantly negated the suppressive impact of irisin on mtROS production and oxidative stress indicators, such as MDA, the GSH/GSSG ratio, CAT activity, and T-SOD levels. (Fig. 7A–F). Additionally, in UCP2-knockdown cells, irisin did not increase Nrf2 or HO-1 expression (Fig. 7G-I), demonstrating that UCP2 is crucial for irisin-mediated activation of the Nrf2 signaling pathway. Moreover, our study indicates that knockdown of Nrf2 also inhibits UCP2 expression (Fig. S7F). This result suggests that Nrf2 regulation may play a critical role in the antioxidant protective mechanism of UCP2, further revealing the mutual regulatory relationship between Nrf2 and UCP2 in the antioxidant effects of irisin.

### UCP2 modulates AMPK activity influencing the Nrf2-HO-1 pathway

**Fig. 8:**
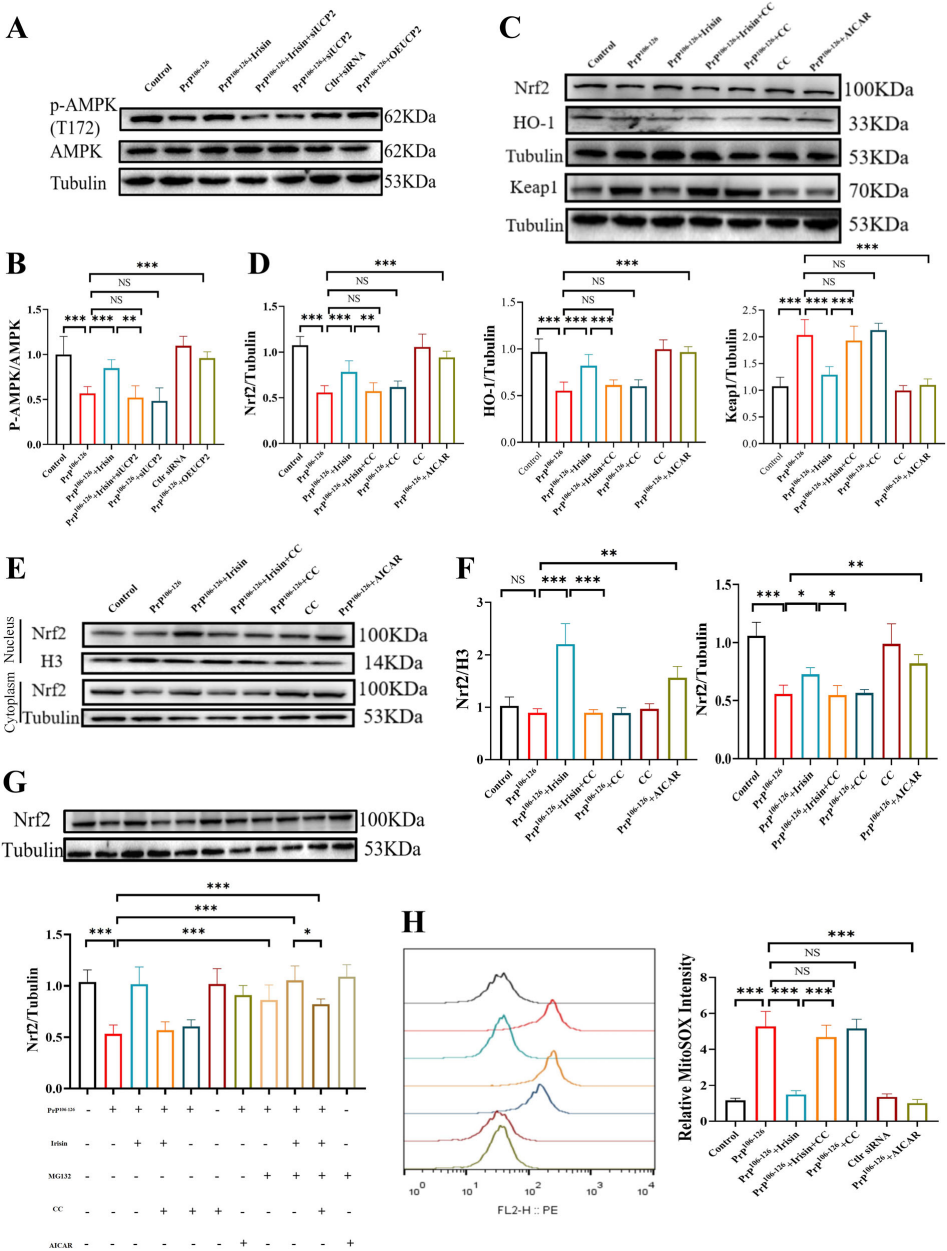
UCP2 modulates AMPK activity, influencing the Nrf2-HO-1 pathway. **A**, **B** The protein expression levels of p-AMPK in N2a cells. **C**, **D** The protein expression levels of Nrf2, HO-1 and Keap1 in N2a cells. **E**, **F** The Nrf2 protein expression in cytosolic and mitochondrial extracts in N2a cells. **G**, **H** Western blot analysis of Nrf2 protein expression in N2a cells treated with PrP^106–126^, irisin, MG132 (proteasome inhibitor), Compound C (AMPK inhibitor), or AICAR (AMPK activator). The data are presented as the means ± SDs (n = 6), *P < 0.05; **P < 0.01; ***P < 0.001.

UCP2 functions as a mild uncoupler in the mitochondrial electron transport chain, effectively reducing the production of ROS [27]. Previous research has demonstrated that UCP2 can activate AMPK [14], a critical regulator of cellular homeostasis during energy stress, and modulate the cellular antioxidant system [28]. Building on these insights, we sought to determine whether AMPK is involved in the mechanism by which irisin mitigates oxidative stress in PrP^106–126^-treated N2a cells. Our observations showed that both irisin pretreatment and UCP2 overexpression increased AMPK phosphorylation levels in N2a and SH-SY5Y cells (Fig. S8A, B), whereas UCP2 silencing abrogated the activation of AMPK by irisin (Fig. 8A, B). Interestingly, irisin did not alter the expression or activation of other key signaling pathways involved in cellular processes, such as AKT, which regulates cell growth and survival, and ERK, which controls cell proliferation and differentiation (Fig. S8C). Irisin treatment significantly reduced Keap1 expression, which normally sequesters Nrf2 and promotes its degradation. This reduction in Keap1 led to an increase in Nrf2 and HO-1 expression, as well as Nrf2 nuclear translocation. To explore the role of AMPK in this process, we treated cells with Compound C (CC), an AMPK inhibitor. (Fig. S8D). The results showed that inhibition of AMPK phosphorylation not only blocked the irisin-induced upregulation of Nrf2 and HO-1 expression but also inhibited Nrf2 nuclear translocation, suggesting that AMPK activity plays a critical role in irisin-mediated Nrf2 activation (Fig. 8C–F).

To further investigate Nrf2 stability, we used the proteasome inhibitor MG132 to block Nrf2 degradation. MG132 treatment alone increased Nrf2 levels in cells co-treated with PrP^106–126^, confirming its effect on Nrf2 stabilization. When cells were co-treated with irisin and MG132, Nrf2 levels were further elevated, suggesting that irisin may promote Nrf2 accumulation by inhibiting its proteasomal degradation or enhancing its stability. However, the increase in Nrf2 levels was reduced upon AMPK inhibition with CC, reinforcing the role of AMPK in stabilizing Nrf2 during irisin treatment (Fig. 8G). Furthermore, in the presence of AMPK inhibition, irisin also failed to maintain its ability to suppress mtROS levels (Fig. 8H). These results underscore that UCP2 and AMPK play a crucial antioxidative protective role in the PrP^106–126^-induced oxidative stress response, specifically through the activation of the Nrf2-HO-1 pathway mediated by AMPK activity. Moreover, irisin stabilizes Nrf2 through the synergistic effects of proteasome inhibition and AMPK activation, thereby enhancing the cell’s antioxidative stress.

## Discussion

Prion diseases are a group of fatal neurodegenerative disorders characterized by the accumulation of misfolded prion proteins, with oxidative stress and mitochondrial damage being common modes of neurotoxic injury [19, 29]. Our research indicated that exogenous administration of irisin can alleviate PrP^106–126^-induced apoptosis in N2a cells and SH-SY5Y cells. This protective effect may be achieved by mitigating oxidative stress, improving mitochondrial function, and enhancing the activity of the electron transport chain. Specifically, in our study, exogenous irisin protected against mitochondrial damage caused by oxidative stress via the UCP2-AMPK signaling pathway, which is crucial for the treatment of prion diseases.

Irisin is a myokine predominantly secreted by skeletal muscles during physical activity, with concentrations in the bloodstream ranging from a few ng/mL to several tens of ng/mL [30]. More importantly, irisin has been found to cross the blood-brain barrier (BBB) and accumulate in neural tissues, where it exerts antioxidant and anti-inflammatory effects [18, 31]. Studies have also shown that irisin regulates glucose and lipid metabolism and exhibits antioxidant effects in type 2 diabetes [17, 32]. In models of neurodegenerative diseases such as Alzheimer’s disease (AD) and Parkinson’s disease (PD), irisin levels are typically lower, and exogenous administration of irisin has been shown to significantly reduce oxidative stress levels in the brain and enhance mitochondrial function [33, 34]. In addition, Zhang et al. used a concentration of 100 ng/mL in an in vitro Parkinson’s disease model and found significant protective effects against mitochondrial damage, as well as improved neuronal survival [35]. These findings are consistent with our results, where we observed that irisin at 100 ng/mL alleviated PrP^106–126^-induced oxidative stress and mitochondrial dysfunction in N2a cells. This provides a useful reference for potential therapeutic applications of irisin in prion diseases.

In mouse models of PD, exogenous irisin reduces oxidative stress and increases the activity of PGC-1α to promote mitochondrial DNA and protein synthesis to protect mitochondrial function [35]. In addition, irisin can also affect the dynamic balance of mitochondria, including the division and fusion process of mitochondria, which is very important for maintaining the stability of the mitochondrial network and its adaptation to different physiological needs [36]. Furthermore, PrP^106–126^ is known to increase mitochondrial membrane permeability in neuroblastoma cells, leading to cytochrome c release and caspase activation, thus triggering apoptosis [37]. Reducing prion-induced neuronal apoptosis can alleviate the associated neuropathology [38]. Previous research has indicated that exogenous irisin can mitigate hepatic cell apoptosis caused by ischemia/reperfusion (I/R) and reduce the area of liver necrosis [36]; it also reduces neuronal apoptosis in models of intracerebral hemorrhage (ICH) [39]. Our study revealed that exogenous irisin can decrease apoptosis by mitigating the release of cytochrome c and the cleavage of caspases induced by PrP^106–126^, thereby restoring cell viability.

Neuronal apoptosis induced by PrP^106–126^ may result from mitochondrial dysfunction. Mitochondria are organelles that generate energy in cells and are crucial for maintaining neuronal health and function [40]. In prion diseases, neurons exhibit signs of mitochondrial dysfunction, including morphological changes, reduced mitochondrial membrane potential, and decreased ATP production. This dysfunction can lead to the neurodegeneration observed in prion diseases, as it causes energy deficits, cellular damage, and ultimately, neuronal death [41]. Our study revealed that exogenous irisin treatment alleviated PrP^106–126^-induced mitochondrial membrane depolarization and restored changes in mitochondrial morphology. OXPHOS, a fundamental mitochondrial function that combines electron transport with cellular respiration and ATP synthesis, establishes a membrane potential across the mitochondrial inner membrane through electron transport [42]. The activity of the mitochondrial respiratory chain is a direct indicator of mitochondrial health and function [43], and previous reports have shown that irisin can significantly enhance the activity of mitochondrial respiratory chain complex I in Parkinson’s disease models to promote ATP production [35]. Therefore, our research also focused on the impact of irisin treatment on the activity of the respiratory chain complexes. The respiratory chain, or electron transport chain, is crucial for cellular energy production. Our study showed that irisin treatment significantly enhances the activity of respiratory chain complexes I-IV. This correlation underscores the protective mechanisms of irisin in the context of mitochondrial function and its ability to promote mitochondrial respiratory chain complexes, mitigating mitochondrial membrane potential depolarization. Overall, these findings support the role of irisin in alleviating PrP^106–126^-induced apoptosis through its ability to protect mitochondrial function.

In this study, we employed Mito-tempo, a mitochondria-targeted antioxidant drug that is a mimic of superoxide dismutase, and is capable of scavenging superoxide and alkyl radicals [44, 45]. Previous studies have shown increased levels of ROS and lipid peroxidation in the cerebral cortex of sporadic Creutzfeldt-Jakob disease (sCJD) patients and Syrian hamsters infected with prions [46]. Our experiments demonstrated that PrP^106–126^ induces substantial mtROS production in N2a cells, and that increased mtROS exacerbates mitochondrial dysfunction. The targeted clearance of mtROS can alleviate the oxidative stress and mitochondrial dysfunction caused by PrP^106–126^. These results highlight the impact of oxidative stress on the pathogenesis of prion diseases. Oxidative stress is a common factor in almost all neurodegenerative diseases. Although first-line drugs that lower ROS levels have been discovered, to date, the effectiveness of many antioxidants in human trials has not been as significant as that in some animal studies. Future research must further explore into the mechanisms of targeted antioxidant therapy to design more effective treatments. A recent study suggests that irisin can reduce oxidative and nitrosative stress, protecting cardiomyocytes [47]. Our study revealed that exogenous irisin treatment significantly decreased oxidative parameters such as mtROS and MDA while increasing the levels of antioxidants such as CAT, SOD, and GSH. Therefore, irisin may alleviate the mitochondrial dysfunction caused by PrP^106–126^-induced oxidative stress, providing neuronal cell protection.

Mitochondrial uncoupling is a key mechanism for reducing the levels of ROS within mitochondria. UCP2, a transmembrane protein of the mitochondrial anion carrier family situated on the inner mitochondrial membrane, contributes to the suppression of mitochondrial ROS synthesis by facilitating increased electron flux and enhancing respiratory chain activity. Prior research has demonstrated that UCP2 overexpression diminishes ROS production within mitochondria, offering protection to renal tubular epithelial cells against oxidative stress-induced apoptosis [48], whereas UCP2 deficiency exacerbates neuroinflammation in primary microglia under oxidative stress [49]. Our findings indicate that irisin can reduce PrP^106-126^-induced oxidative stress through its interaction with UCP2, and that interference with UCP2 negates the protective effects of irisin. Mitochondrial uncoupling, leading to the decoupling of ATP production from oxidative phosphorylation, serves as a primary mechanism for AMPK activation [50]. AMPK, an evolutionarily conserved kinase, plays a vital role in multiple cellular processes including metabolism, energy homeostasis, cell growth, inflammation, infection response, redox regulation, and tissue repair and regeneration. Consistent with these findings, our study showed that irisin augments AMPK activation in neuroblastoma cells and SH-SY5Y cells treated with PrP^106–126^, and that UCP2 knockdown eliminates this AMPK activation. Moreover, inhibiting AMPK abrogates the reduction in oxidative stress caused by irisin in neuroblastoma cells treated with PrP^106–126^, indicating that the uncoupling-induced AMPK activation is essential for mitigating oxidative stress in PrP^106–126^-treated cells with impaired UCP2 function.

Nrf2 is a transcription factor that upregulates the expression of antioxidant genes, such as HO-1, and is crucial for defending against oxidative stress [51]. AMPK is thought to mediate antioxidative actions by enhancing the transcriptional activity of Nrf2 [52]. Our study revealed that irisin activates AMPK, modulates Nrf2 transcriptional activity, and elevates the expression of the antioxidant enzyme HO-1, thereby mitigating intracellular oxidative stress. These findings not only reveal the potential mechanisms through which irisin regulates mitochondrial function but also emphasize its therapeutic potential in combating neurodegenerative diseases.

Recent studies have shown that AKT and ERK1/2 signaling pathways mediate irisin’s neuroprotective effects in Parkinson’s disease by preventing mitochondrial damage and reducing oxidative stress [35]. In contrast, our investigation in a prion disease model revealed that prion infection altered AKT phosphorylation, while ERK1/2 signaling remained unaffected. Importantly, irisin treatment did not modulate either AKT or ERK1/2 pathways in this model, suggesting that the neuroprotective effects of irisin in prion disease may operate through mechanisms independent of these pathways. This suggests that irisin may exert its effects through different mechanisms in various neurodegenerative diseases. Specifically, in prion disease models, irisin’s neuroprotective effects may rely on the UCP2/AMPK/Nrf2 axis rather than the AKT/ERK1/2 pathway. This raises an important question of how irisin exerts its beneficial effects across different neurodegenerative diseases. Future studies should focus on elucidating the activation mechanisms of different signaling pathways by irisin in various degenerative diseases to uncover new therapeutic strategies.

In summary, our study highlights the vital role of exogenous irisin in regoverning oxidative stress and safeguarding mitochondrial function via the UCP2-AMPK pathway. By facilitating the uncoupling and activation of AMPK, irisin not only decreases oxidative stress and prevents mitochondrial dysfunction but also enhances cellular viability. This finding new avenues and strategies for treating a variety of neurodegenerative conditions, including prion diseases. Further research is warranted to investigate the specific application of these mechanisms across different models and clinical conditions, aiming for more targeted and efficacious treatment modalities.

## Supplementary information

SUPPLEMENTAL MATERIALS

The original Western blot bands

## Data Availability

The data supporting this study can be obtained from the corresponding author upon reasonable request.
