# Supplementary material for: Irisin regulates oxidative stress and mitochondrial dysfunction through the UCP2-AMPK pathway in prion diseases
Source: Cell Death Dis. 2025 Feb 3;16(1):66. doi: 10.1038/s41419-025-07390-w (PMC11790890; doi:10.1038/s41419-025-07390-w)
Supplement: Supplementary file 1 — SUPPLEMENTAL MATERIALS [file 41419_2025_7390_MOESM1_ESM.docx]

**Supplementary materials**

**Supplementary figures**


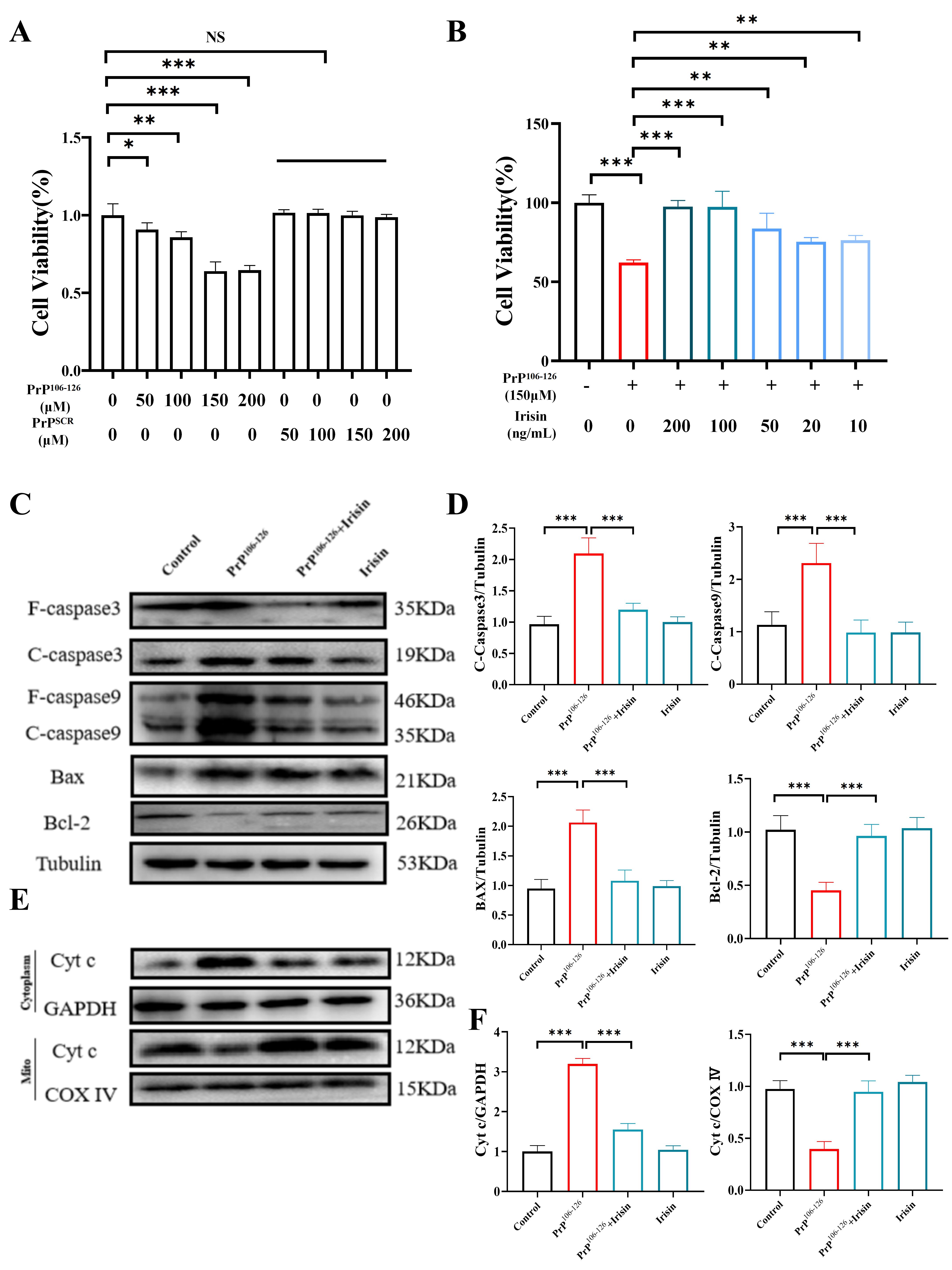


**Figure S1. Irisin Reduces PrP^106-126^-Induced Apoptosis.**

(A) N2a cell viability was measured using CCK8 kit after PrP^106-126^ treatment. (B) N2a cell viability was measured using CCK8 kit after irisin treatment. (C) and (D) Detection of cleaved caspase-3, cleaved caspase-9, Bax and Bcl2 protein expression in SH-SY5Y cells by Western blotting. (E) and (F) The cytochrome c protein expression in cytosolic and mitochondrial extracts in SH-SY5Y cells. The data are presented as the means ± SDs (n=6), **P* < 0.05; ***P* < 0.01; ****P* < 0.001.


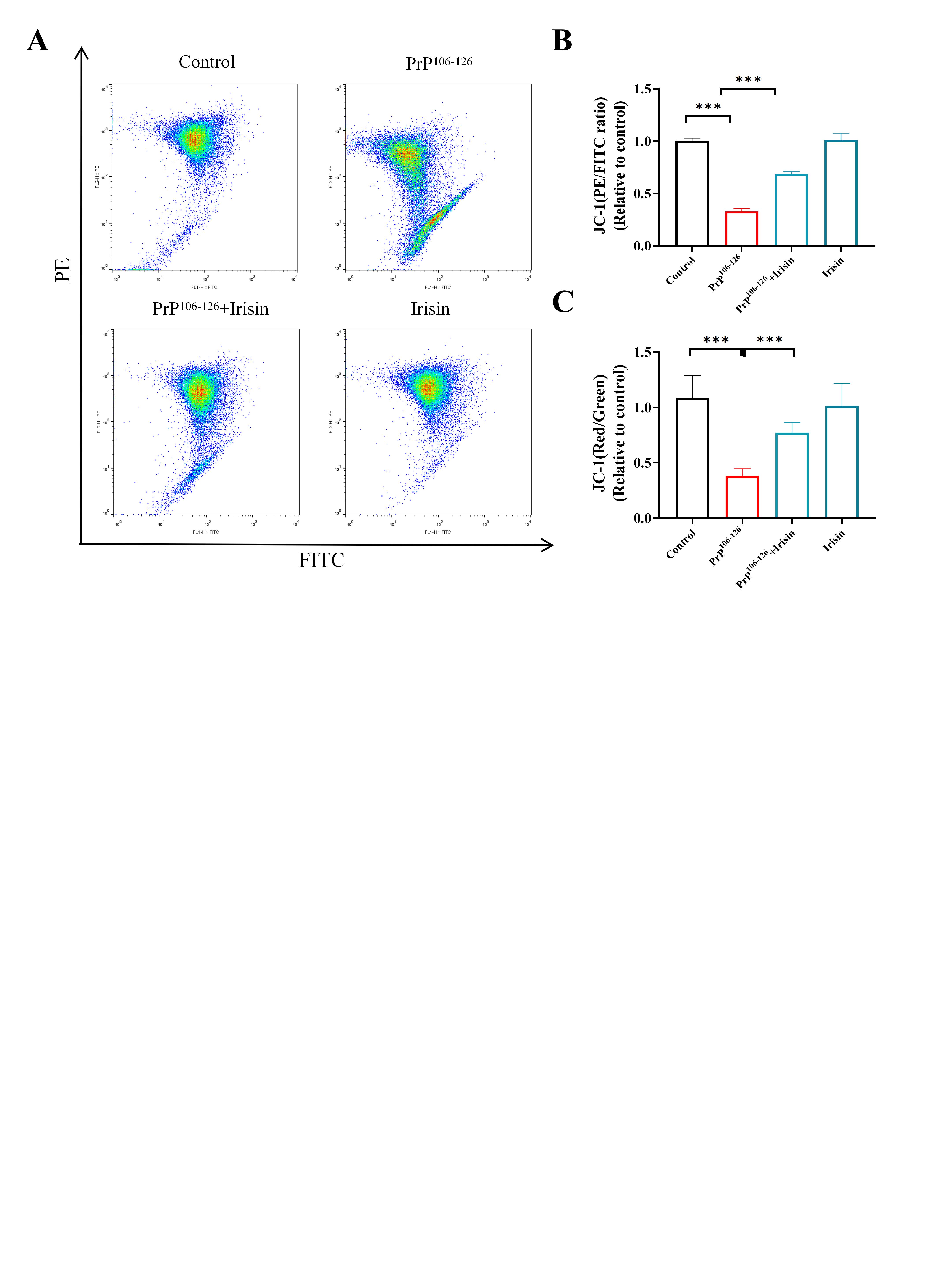


**Figure S2. Effects of Irisin and Mito-Tempo on Mitochondrial Membrane Potential in PrP^106-126^-Treated N2a Cells.**

(A) and (B) Flow cytometry analysis of mitochondrial membrane potential using JC-1 staining in N2a cells treated with PrP^106-126^ and irisin. (C) Quantification of JC-1 staining fluorescence in N2a cells treated with PrP^106-126^ and irisin. Data is presented as the mean ± SD (n=6), ****P* < 0.001.


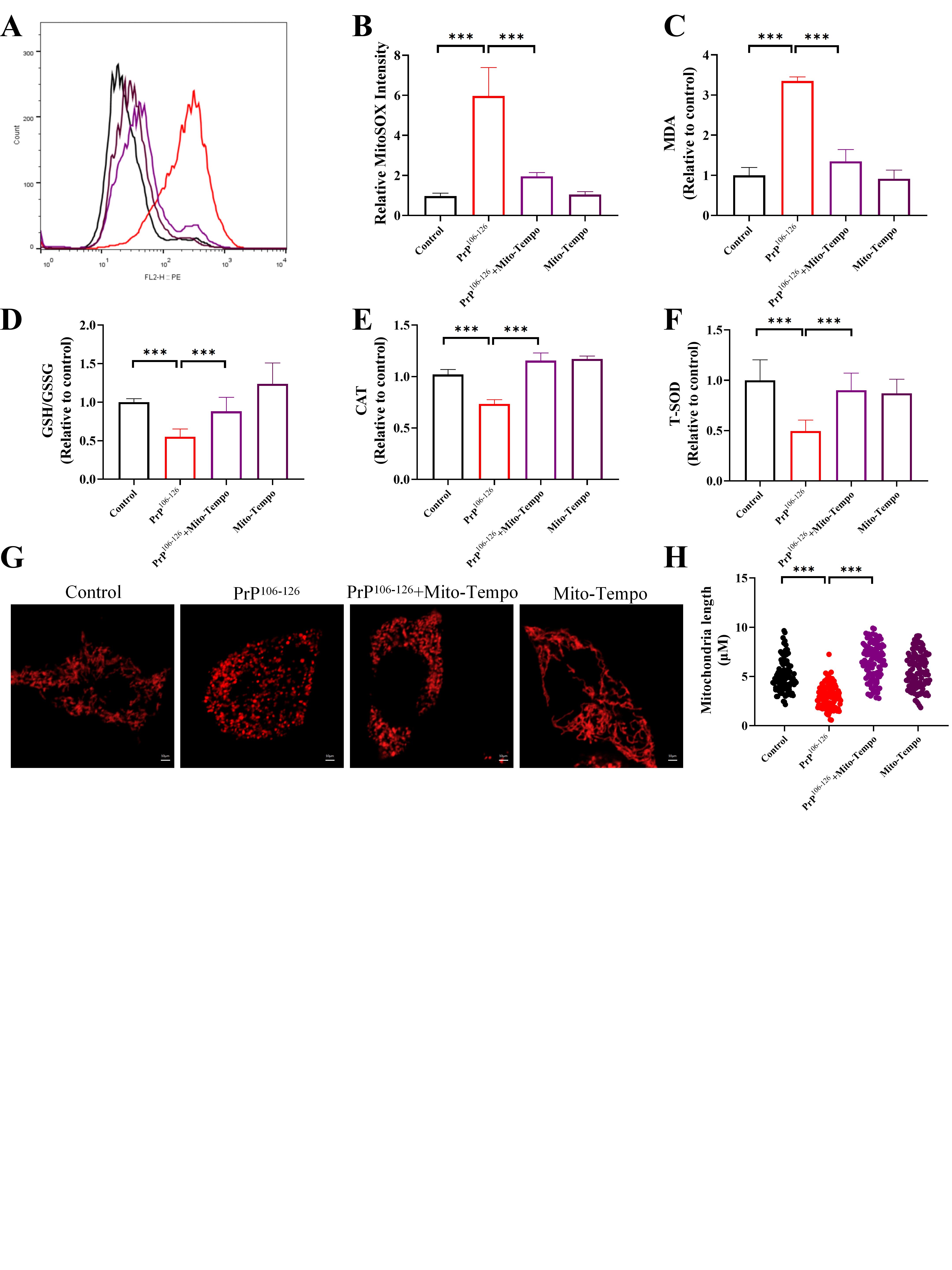


**Figure S3. Irisin Relieves PrP^106-126^-Induced Neuronal Oxidative Stress in N2a Cells**

(A) and (B) Detection of mtROS production in N2a cells treated with Mito-Tempo and PrP^106-126^, analyzed using MitoSOX staining and flow cytometry. (C-F) Measurement of MDA content, GSH/GSSH ratio, and CAT and T-SOD activities in N2a cells. (G) and (H) The representative images of mitochondrial morphology and the quantification of mitochondrial length (scale bar = 10 µm). ****P* < 0.001.

**
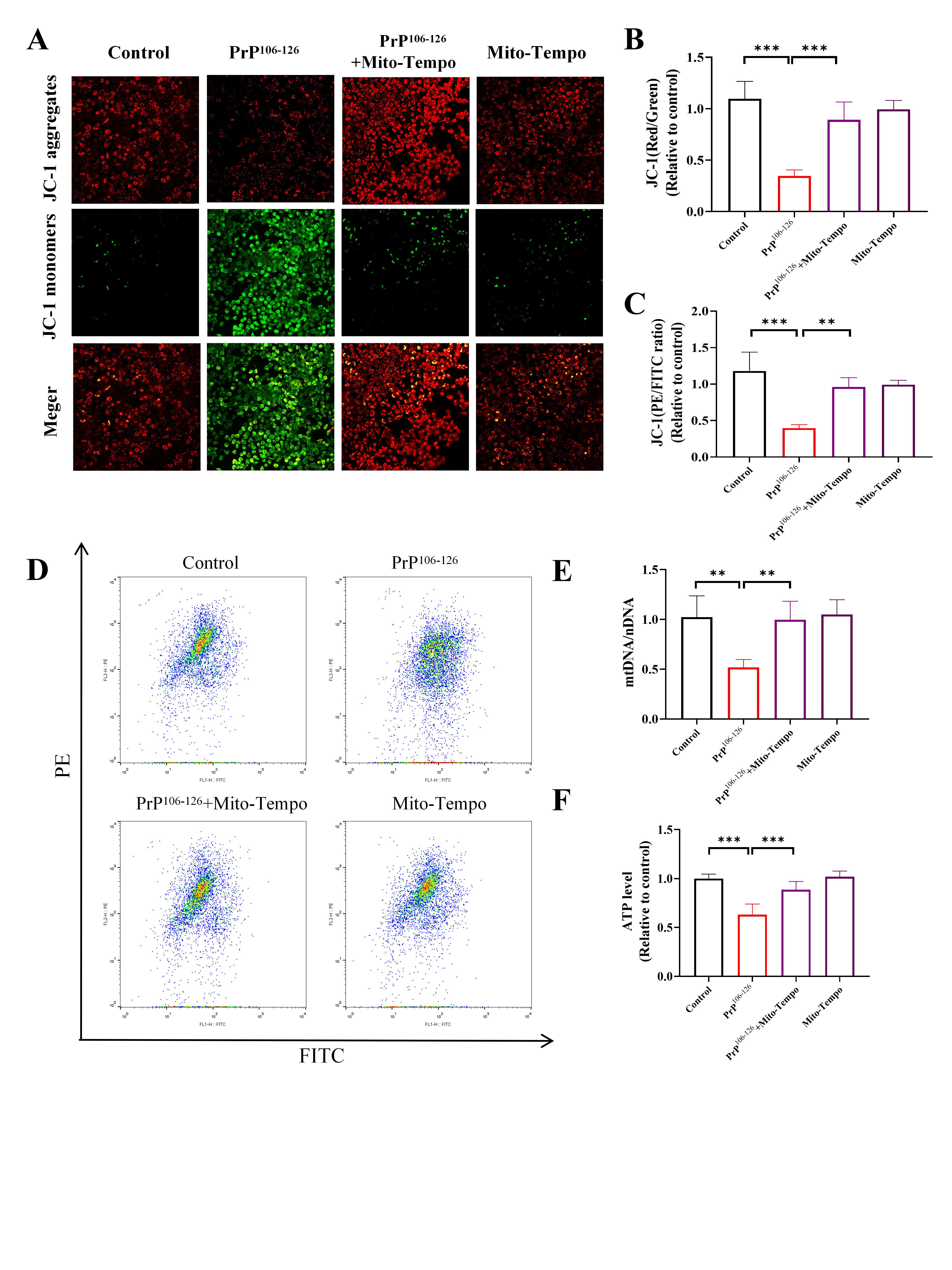
**

**Figure S4. Irisin Relieves PrP^106-126^-Induced Neuronal Oxidative Stress in N2a Cells.**

(A-D) Mitochondrial membrane potential (MMP) measurement by JC-1 dye in N2a cells (scale bar = 50 µm). (E) The mtDNA/nDNA ratio was assessed by real-time PCR. (F) Measurement of ATP levels in N2a cells. The data are presented as the means ± SDs (n=6), ***P* < 0.01; ****P* < 0.001.


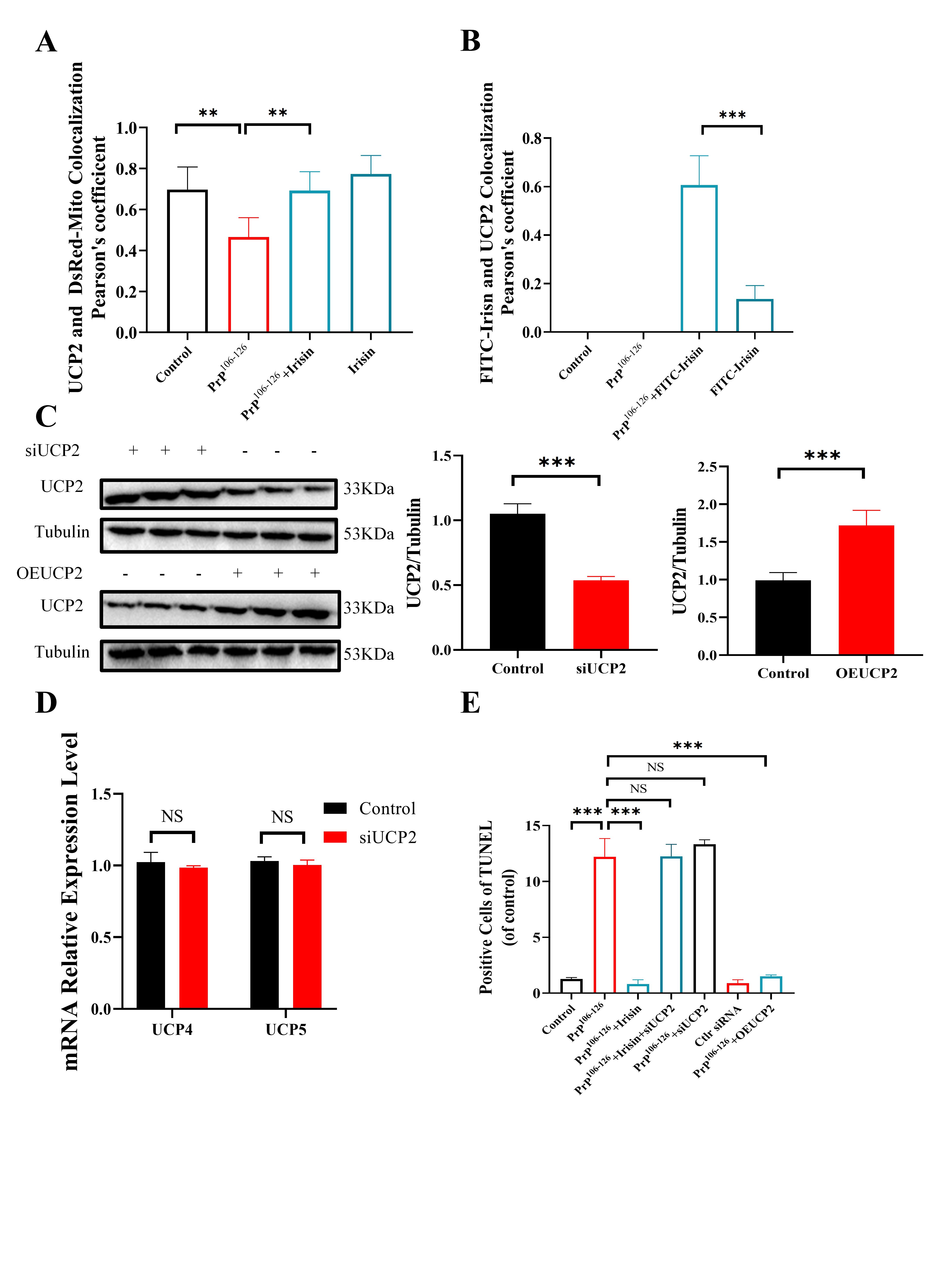


**Figure S5. Effects of Irisin and UCP2 on Mitochondrial Membrane Potential and Antioxidant Pathways in PrP^106-126^-Treated N2a Cells.**

(A)Quantification of UCP2 and mitochondrial marker DsRed-Mito colocalization in N2a cells treated with PrP^106-126^ and irisin. (B) Quantification of FITC-Irisin and UCP2 colocalization in N2a cells treated with PrP^106-126^ and irisin. (C) Western blot analysis of UCP2 expression following siRNA knockdown (siUCP2) and overexpression (OEUCP2) treatments in N2a cells. (D) Relative mRNA expression levels of UCP2 and UCP5 in N2a cells treated with siUCP2. (E) Quantification of TUNEL-positive cells in N2a cells. Data is presented as the mean ± SD (n=6), ***P* < 0.01; ****P* < 0.001.


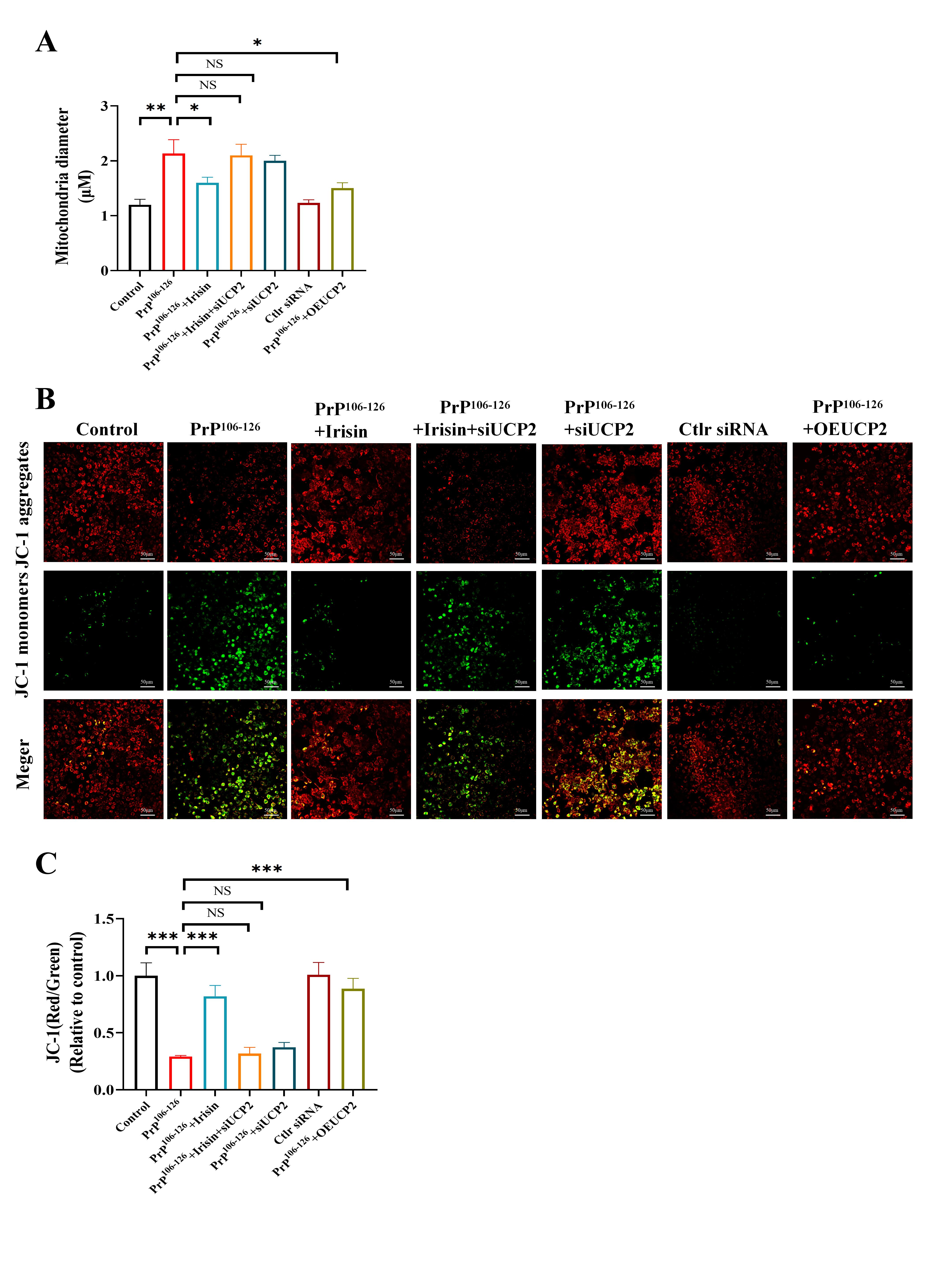


**Figure S6. Effects of Irisin on Mitochondrial Morphology and Membrane Potential in PrP^106-126^-Treated N2a Cells.**

(A) Quantification of mitochondrial diameter in N2a cells. (B)and (C) Representative JC-1 fluorescence images and quantification in PrP^106-126^-treated N2a cells after irisin treatment and either UCP2 knockdown or overexpression. Data is presented as the mean ± SD (n=6), **P* < 0.05; ***P* < 0.01; ****P* < 0.001.


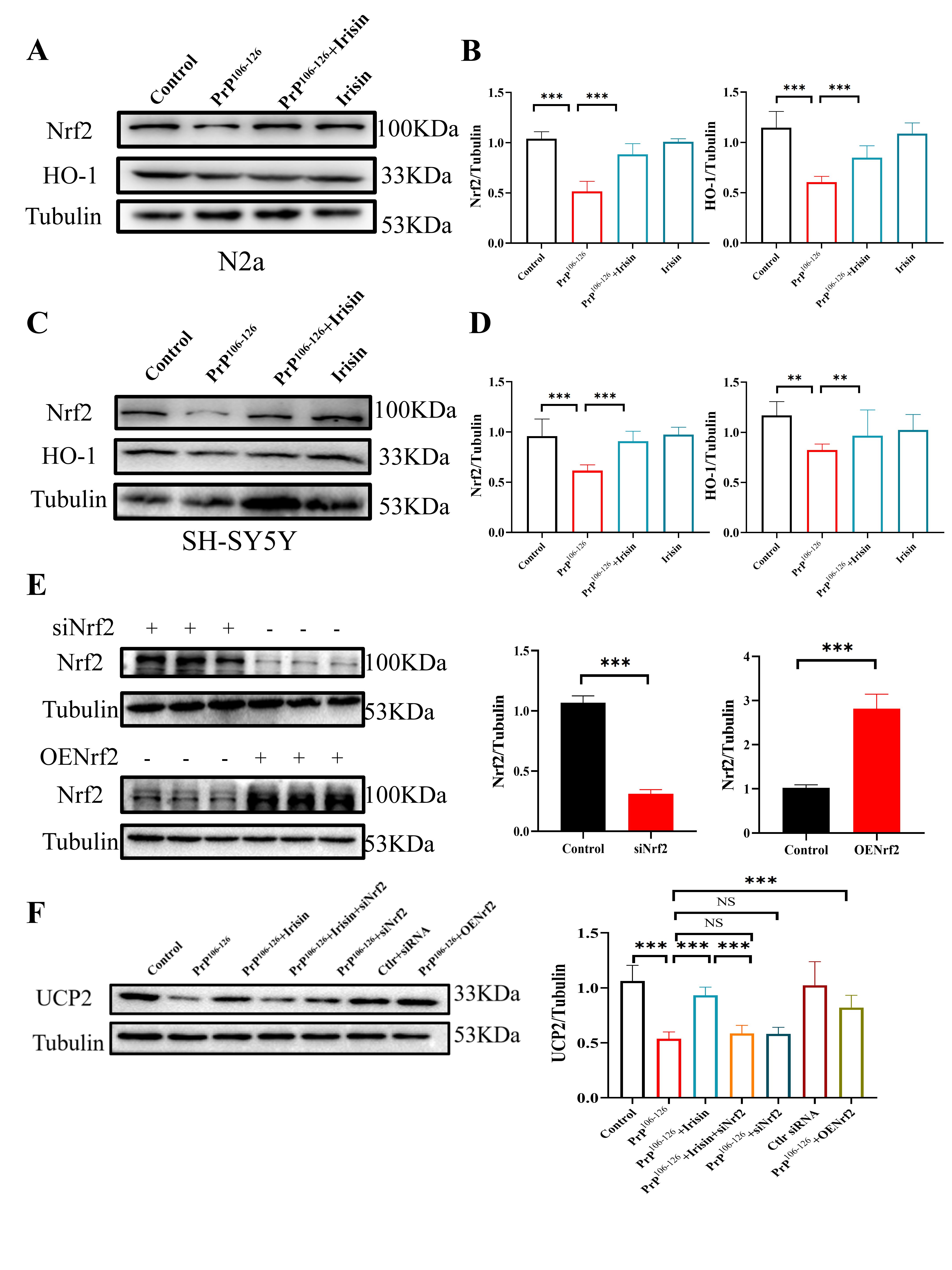


**Figure S7. Effects of Irisin Against PrP^106-126^-Induced Oxidative Damage Through the Nrf2/HO-1 and UCP2 Pathways**

1. and (B) Detection of Nrf2 and HO-1 protein expression changes in N2a cells by Western blotting. (C) and (D) Detection of Nrf2 and HO-1 protein expression changes in SH-SY5Y cells by Western blotting. (E) Western blot analysis of Nrf2 expression under conditions of Nrf2 knockdown (siNrf2) and overexpression (OENrf2) in N2a cells. (F) Western blot analysis of UCP2 protein levels in PrP^106-126^-treated N2a cells after irisin treatment and either Nrf2 knockdown or overexpression. Data is presented as the mean ± SD (n=6), ***P* < 0.01; ****P* < 0.001.


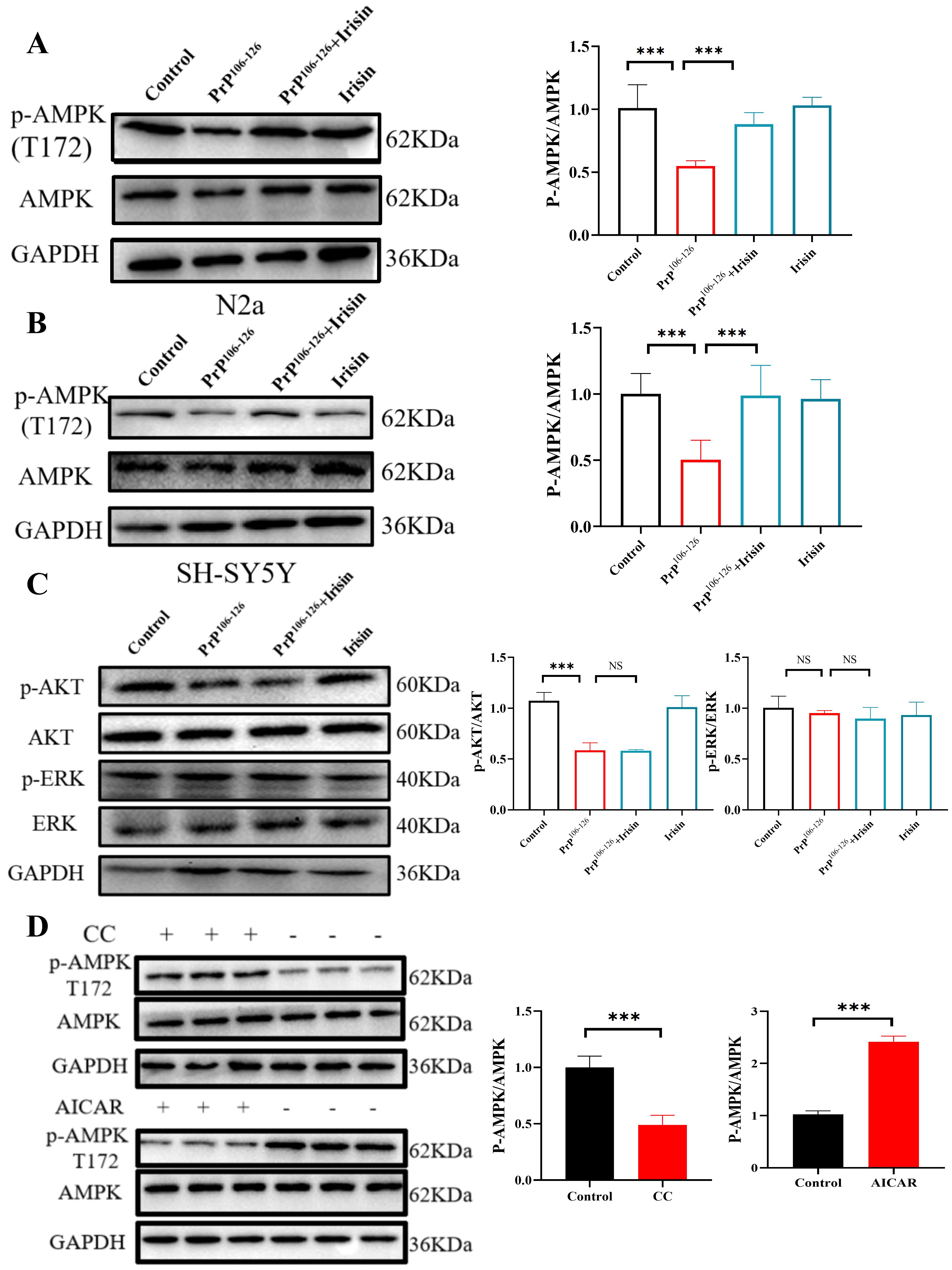


**Figure S8. Irisin Ameliorates PrP^106-126^-Induced Oxidative Damage Through the AMPK Pathway.**

1. Detection of p-AMPK protein expression changes in N2a cells by Western blotting. (B) Detection of p-AMPK protein expression changes in SH-SY5Y cells by Western blotting. (C) Western blot analysis of p-AKT and p-ERK in N2a cells treated with PrP^106-126^ and irisin. (D) Western blot analysis of p-AMPK expression under conditions of CC and AICAR in N2a cells. Data is presented as the mean ± SD (n=6), ****P* < 0.001.

**Table S1. The primer sequences applied for RT-qPCR**

| **Gene name** | **Forword sequences (5'-3')** | **Reverse sequences (5'-3')** |
| --- | --- | --- |
| mtDNA | CCTATCACCCTTGCCATCAT | GAGGCTGTTGCTTGTGTGTGAC |
| gDNA | ATGGAAAGCCTGCCATCATG | TCCTTGTTGTTCAGCATCAC |
| UCP2 | TAAAGGTCCGCTTCCAGGCTCA | TAAAGGTCCGCTTCCAGGCTCA |
| UCP4 | TCTACGGGAAGTCGTGTTTGGC | AACTGTCCGATGACACCAGCCA |
| UCP5 | GTGCTGCAATCGTTGTGGGAGT | GCCAAACCACAGGTGAAACTGG |
| GAPDH | CATCACTGCCACCCAGAAGACTG | ATGCCAGTGAGCTTCCCGTTCAG |

**Table S2. Antibody for western blot**

| **Antigen** | **Host** | **Dilution** | **Cat number** | **Source** |
| --- | --- | --- | --- | --- |
| caspase 3 | rabbit | 1:1000 | 19677-1-AP | Proteintech |
| caspase 9 | rabbit | 1:1000 | 10380-1-AP | Proteintech |
| Bax | rabbit | 1:1000 | 50599-2-Ig | Proteintech |
| bcl2 | rabbit | 1:1000 | 68103-1-Ig | Proteintech |
| cytochrome c | rabbit | 1:3000 | 66264-1-Ig | Proteintech |
| UCP2 | rabbit | 1:1000 | 11081-1-AP | Abcam |
| p-AMPK(T172) | rabbit | 1:1000 | 2535 | CST |
| AMPK | rabbit | 1:1000 | 5831 | CST |
| HO-1 | rabbit | 1:1000 | 66743-1-Ig | Proteintech |
| Nrf2 | rabbit | 1:1000 | 16396-1-AP | Proteintech |
| Keap1 | rabbit | 1:1000 | 10503-2-AP | Proteintech |
| COXIV | rabbit | 1:1000 | 11242-1-AP | Proteintech |
| p-AKT | rabbit | 1:1000 | 4060 | CST |
| AKT | rabbit | 1:1000 | 9272 | CST |
| p-ERK | mouse | 1:1000 | 4370 | CST |
| ERK | mouse | 1:1000 | 83533-1-RR | Proteintech |
| H3 | rabbit | 1:5000 | 17168-1-AP | Proteintech |
| α-tubulin | rabbit | 1:10000 | 11224-1-AP | Proteintech |
| GAPDH | mouse | 1:10000 | 66004-1-lg | Proteintech |
| HRP-conjugated IgG | mouse | 1:10000 | ZB-2305 | ZsBio |
| HRP-conjugated IgG | rabbit | 1:10000 | ZB-2301 | ZsBio |
