# Supplementary material for: Irisin regulates oxidative stress and mitochondrial dysfunction through the UCP2-AMPK pathway in prion diseases
Source: Cell Death Dis. 2025 Feb 3;16(1):66. doi: 10.1038/s41419-025-07390-w (PMC11790890; doi:10.1038/s41419-025-07390-w)

Figure1-E(A : Control; B : PrP106-126; C :Irisin+PrP106-126; D : Irisin)

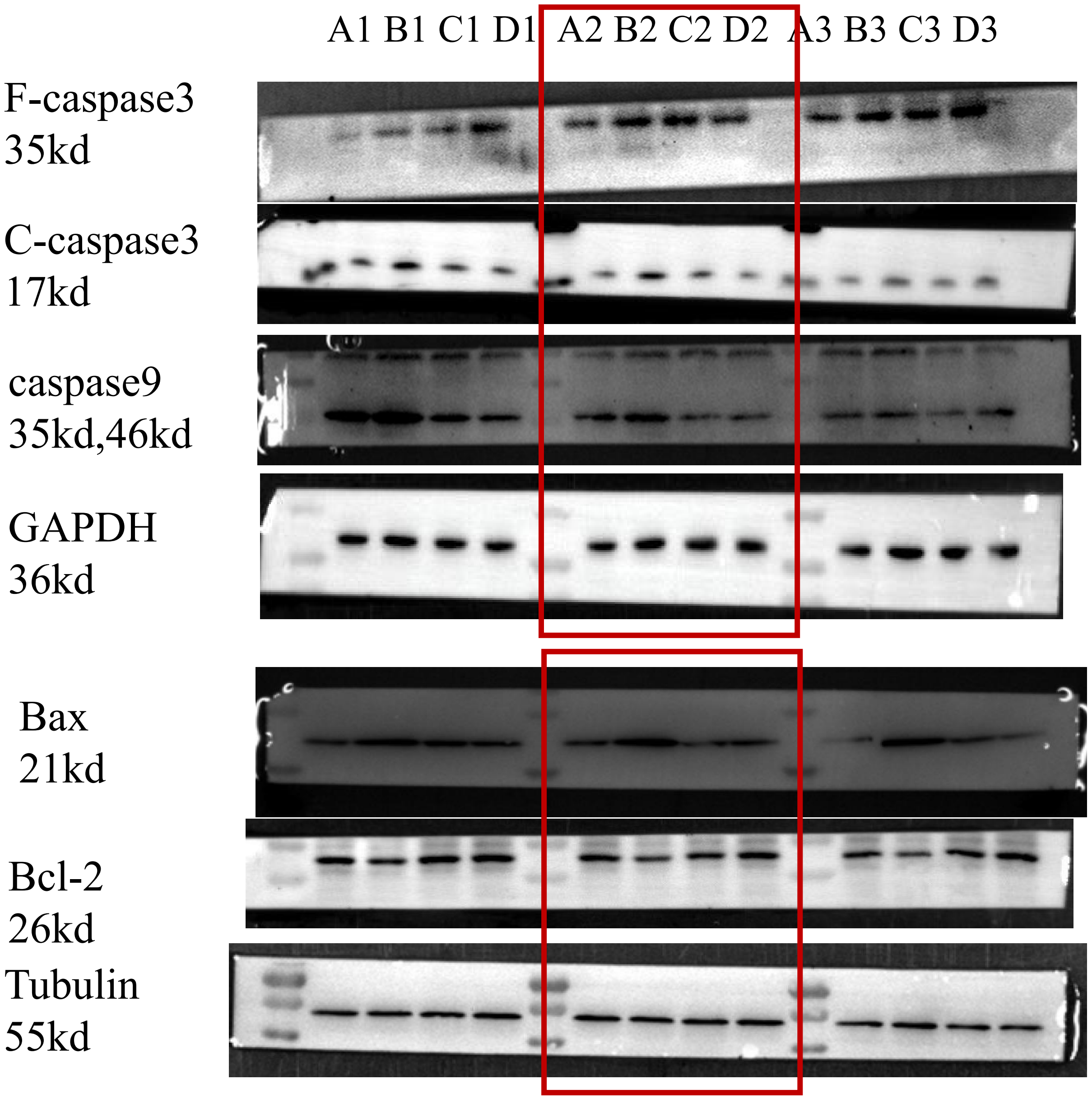

Figure1-J(A : Control; B : PrP106-126; C :Irisin+PrP106-126; D : Irisin)

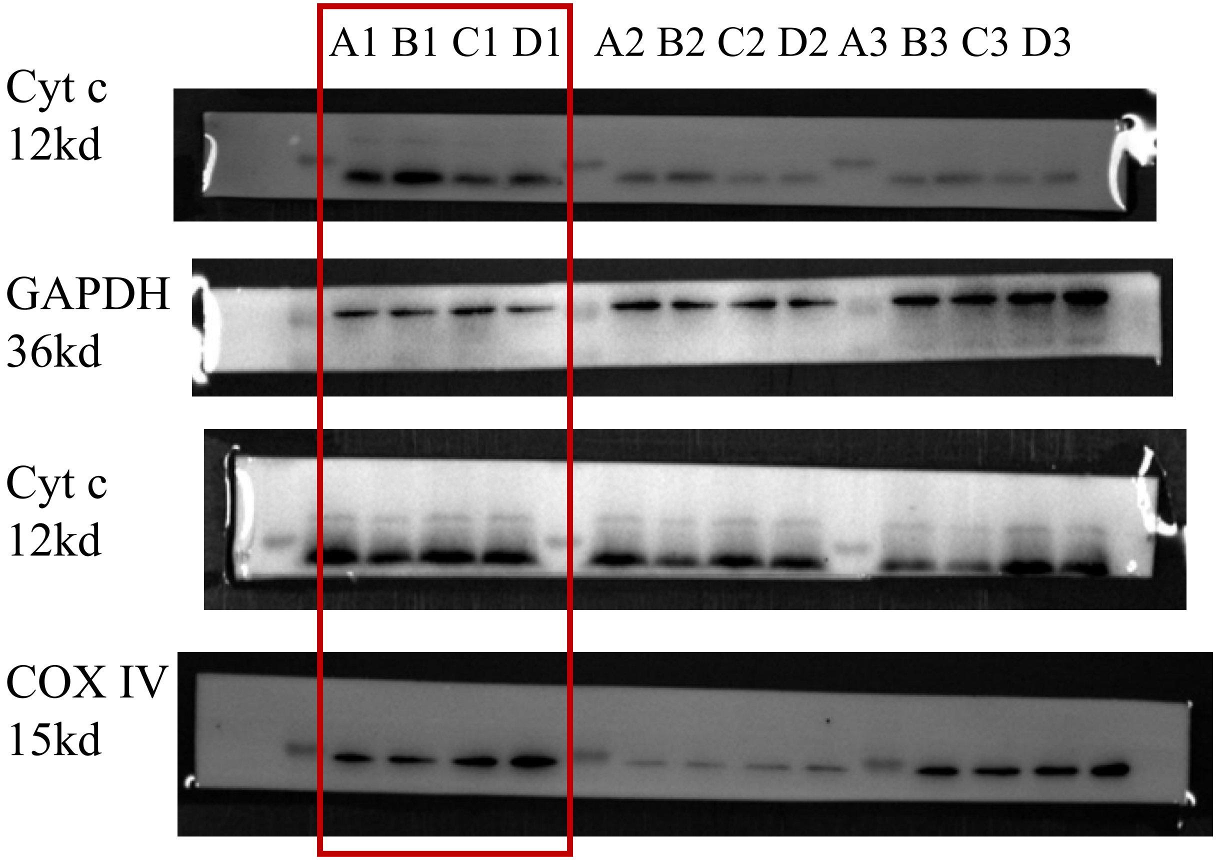

Figure4-E(A : Control; B : 3h; C :6h; D : 12h; E: 24h； E: 36h)

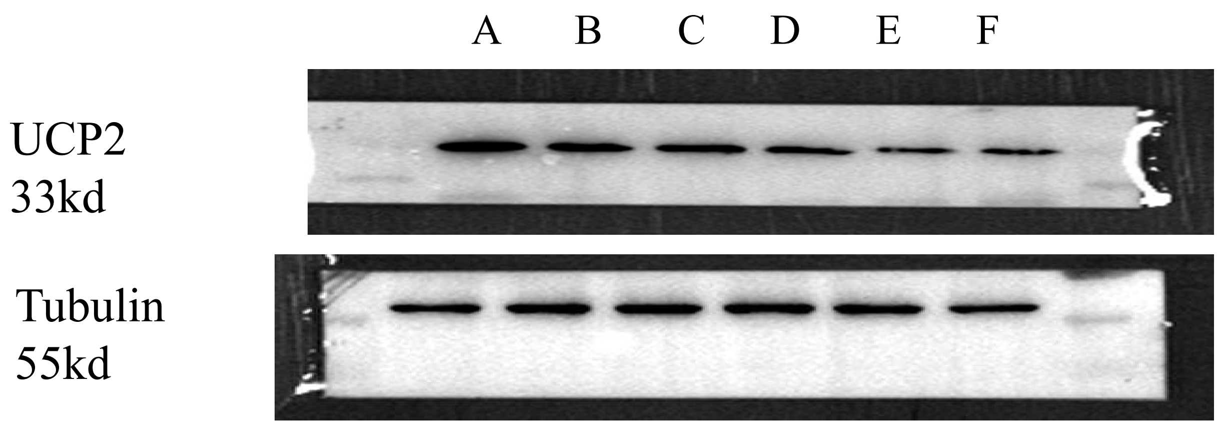

Figure4-F(A : Control; B : PrP106-126; C :Irisin+PrP106-126; D : Irisin)

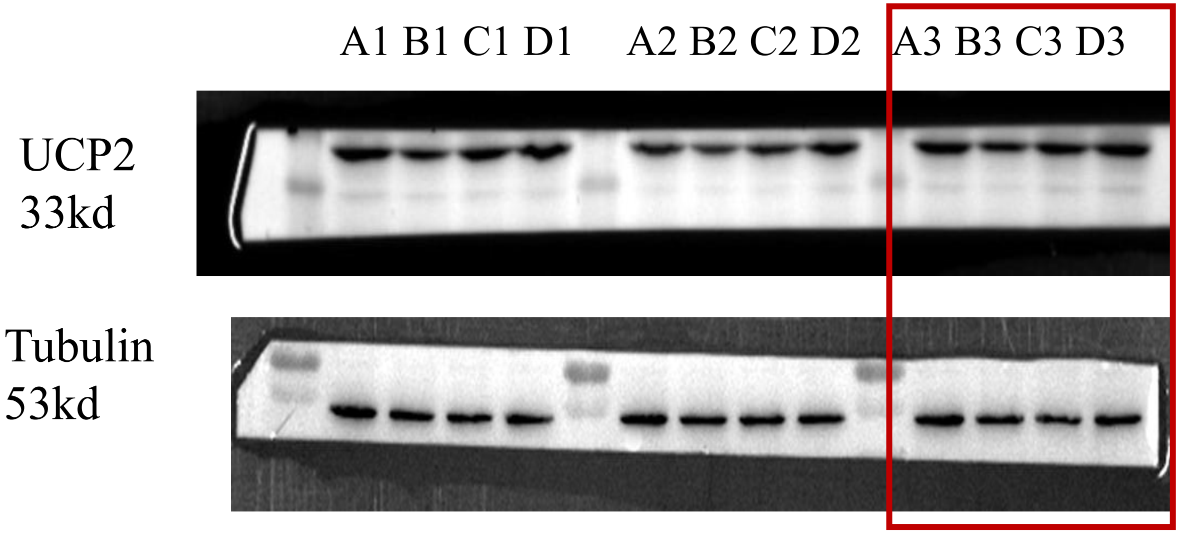

Figure7-G (A : Control; B : PrP106-126; C :Irisin+PrP106-126;
D : PrP106-126+Irisin+siUCP2; E : PrP106-126+siUCP2;
F: Ctlr siRNA; G:PrP106-126+OEUCP2)

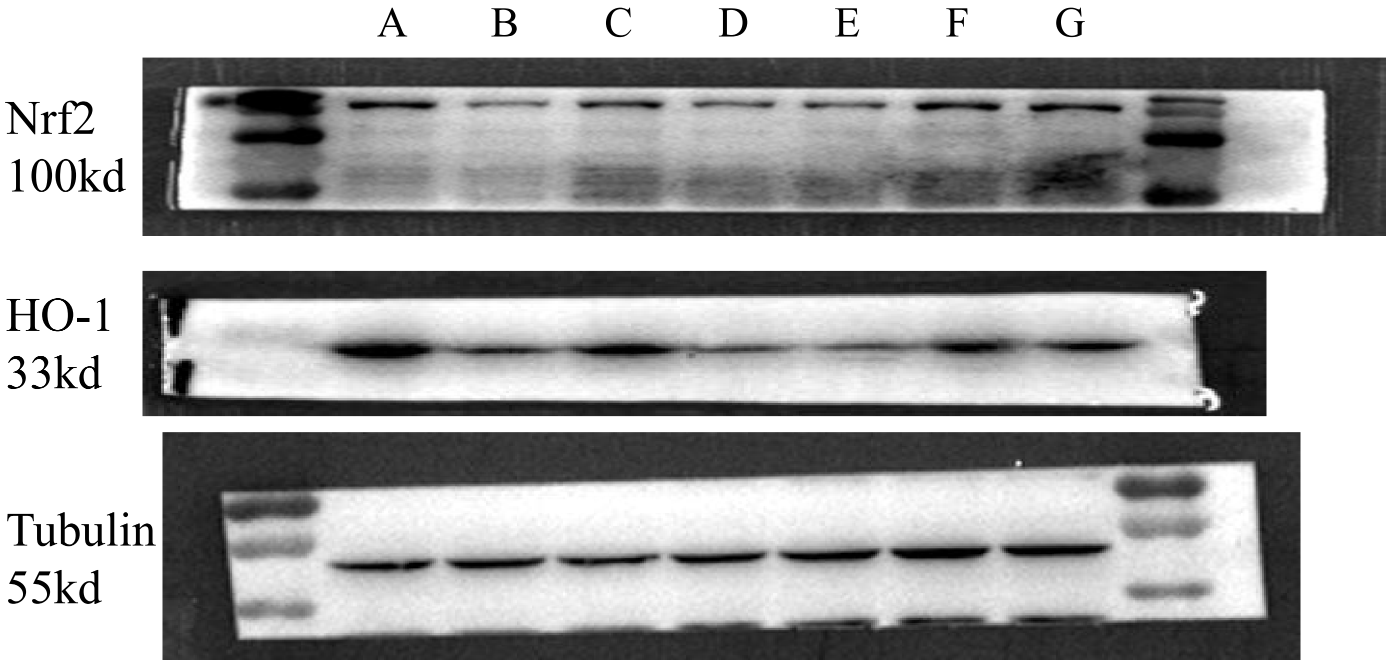

Figure8-A (A : Control; B : PrP106-126; C :Irisin+PrP106-126; D : PrP106-126+Irisin+siUCP2; E : PrP106-126+siUCP2; F: Ctlr siRNA; G:PrP106-126+OEUCP2)

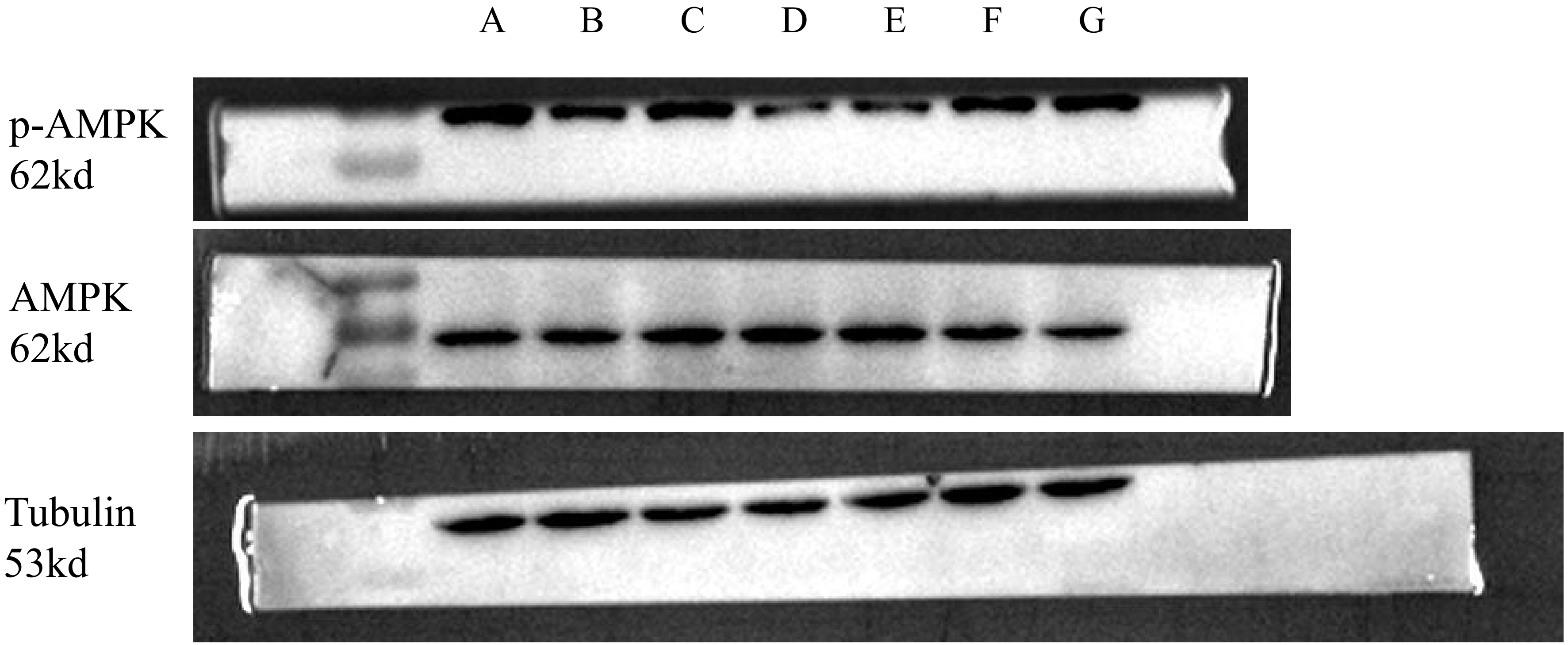

Figure8-C (A : Control; B : PrP106-126; C :Irisin+PrP106-126;
D : PrP106-126+Irisin+CC; E : PrP106-126+CC;
F: CC; G:PrP106-126+AICAR)

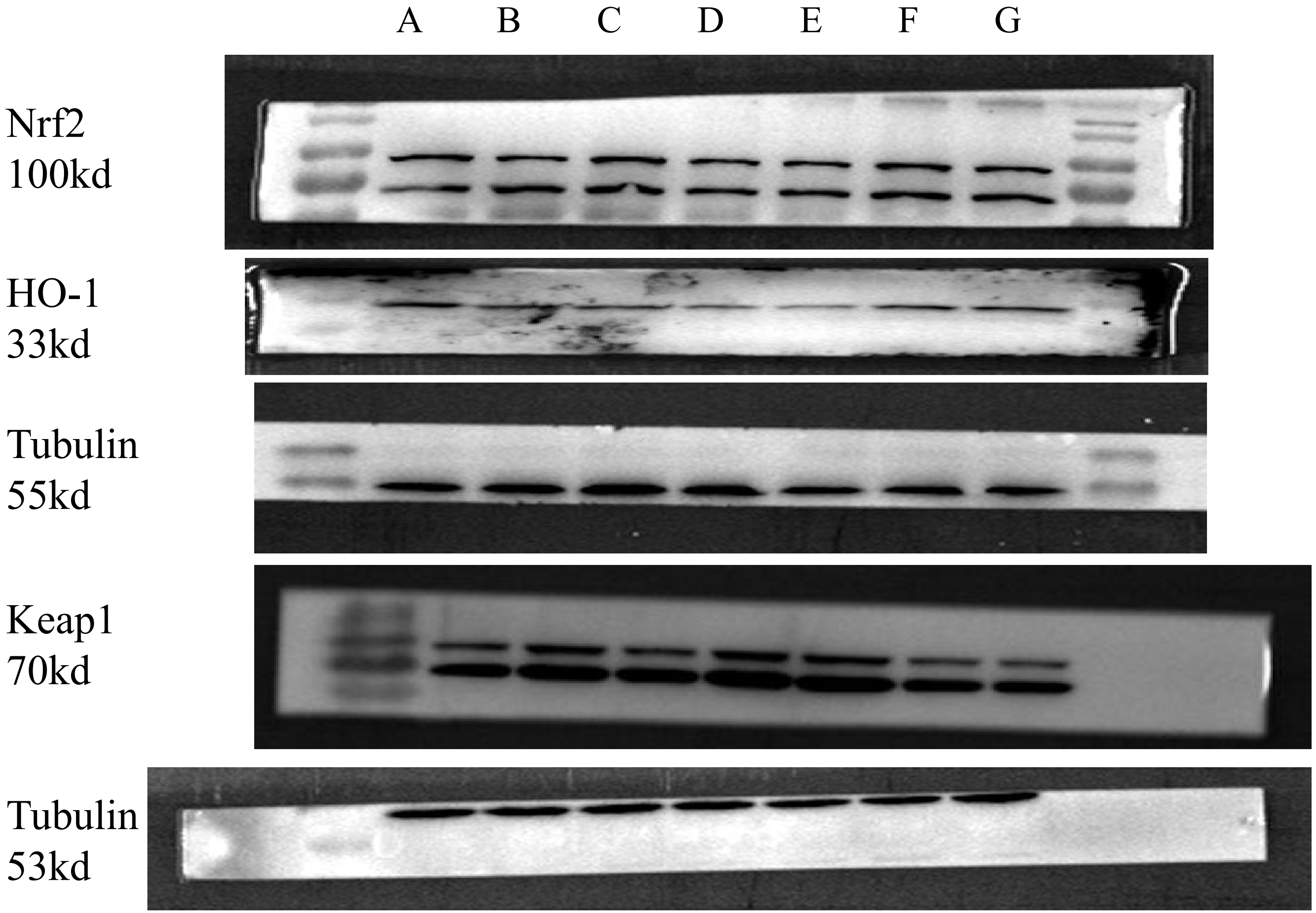

Figure8-E (A : Control; B : PrP106-126; C :Irisin+PrP106-126; D : PrP106-126+Irisin+CC; E : PrP106-126+CC; F: CC; G:PrP106-126+AICAR)

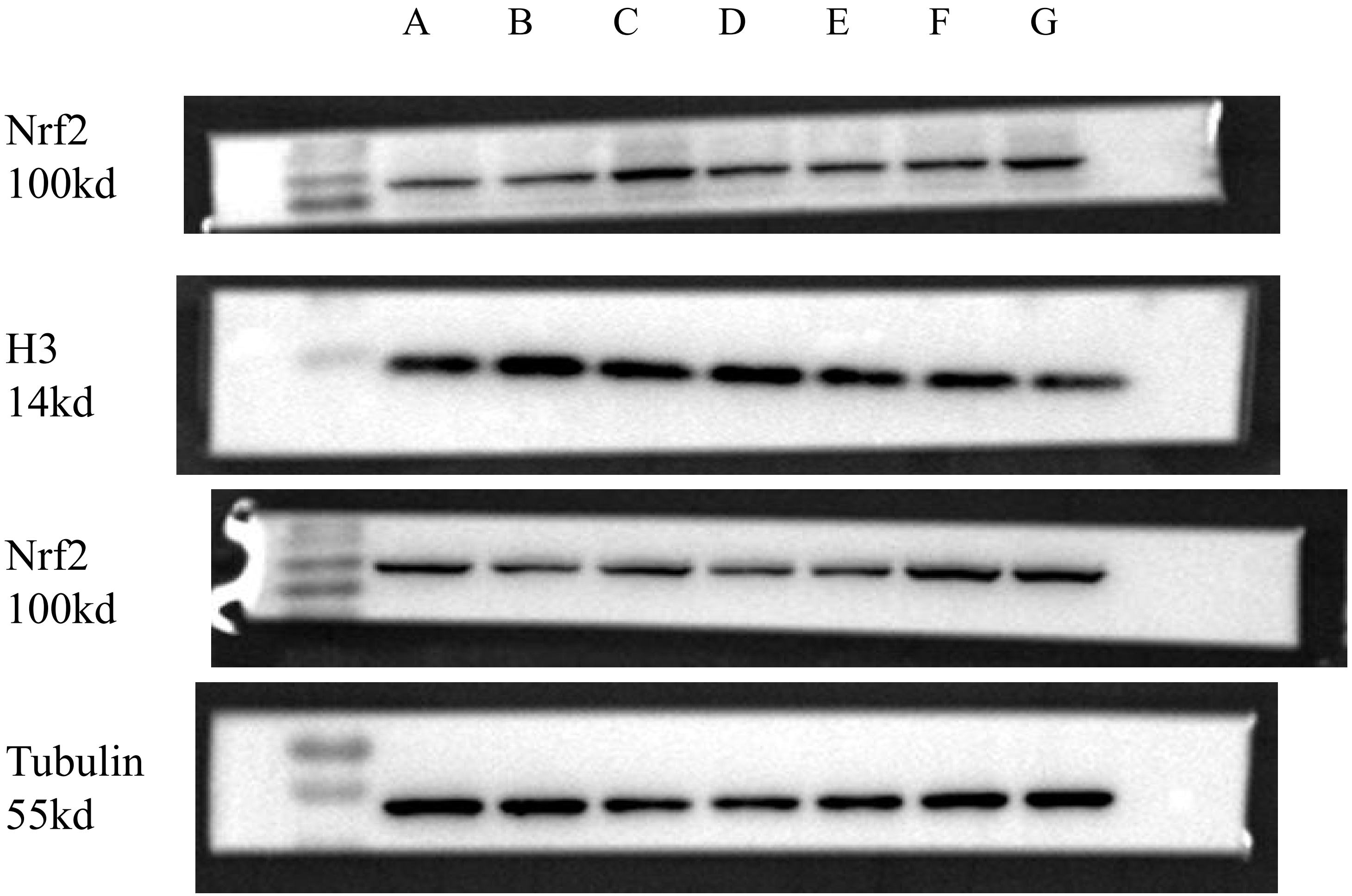

Figure8-G (A：Control；B : PrP106-126；C：Irisin+PrP106-126；
D：PrP106-126+Irisin+CC；E：PrP106-126+CC；
F：CC; G:PrP106-126+AICAR；H：PrP106-126+MG132；
I：PrP106-126+Irisin+MG132；J：PrP106-126+Irisin+MG132+CC;
K：MG132

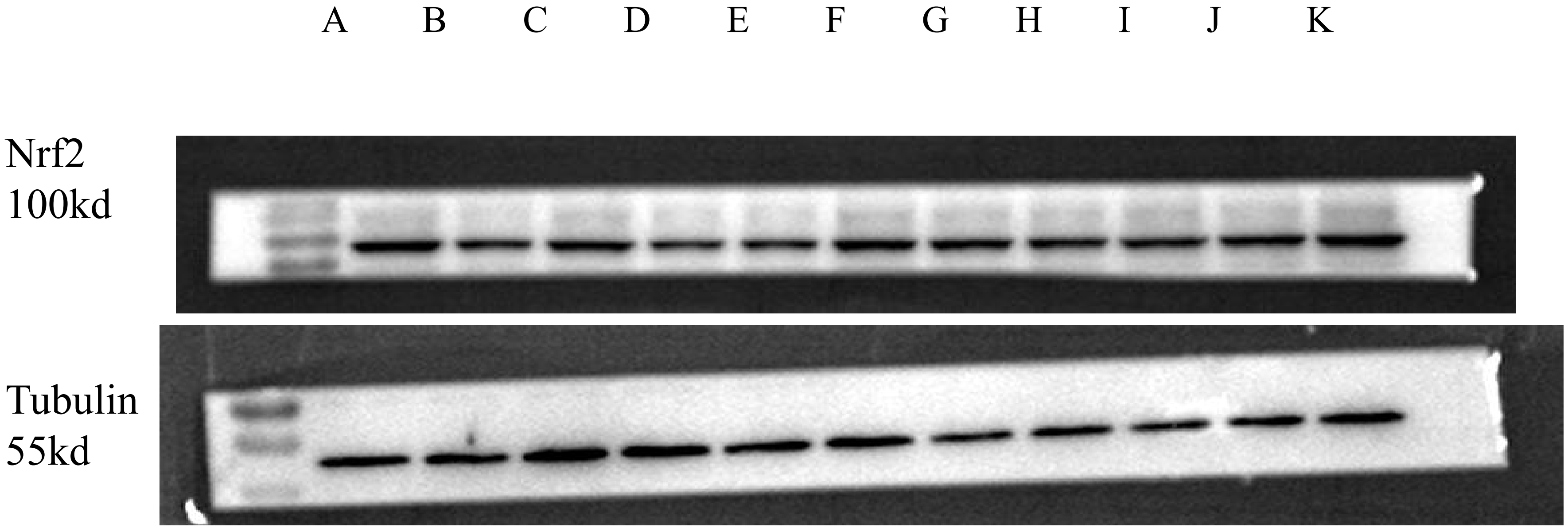

FigureS1(A : Control; B : PrP106-126; C :Irisin+PrP106-126; D : Irisin)

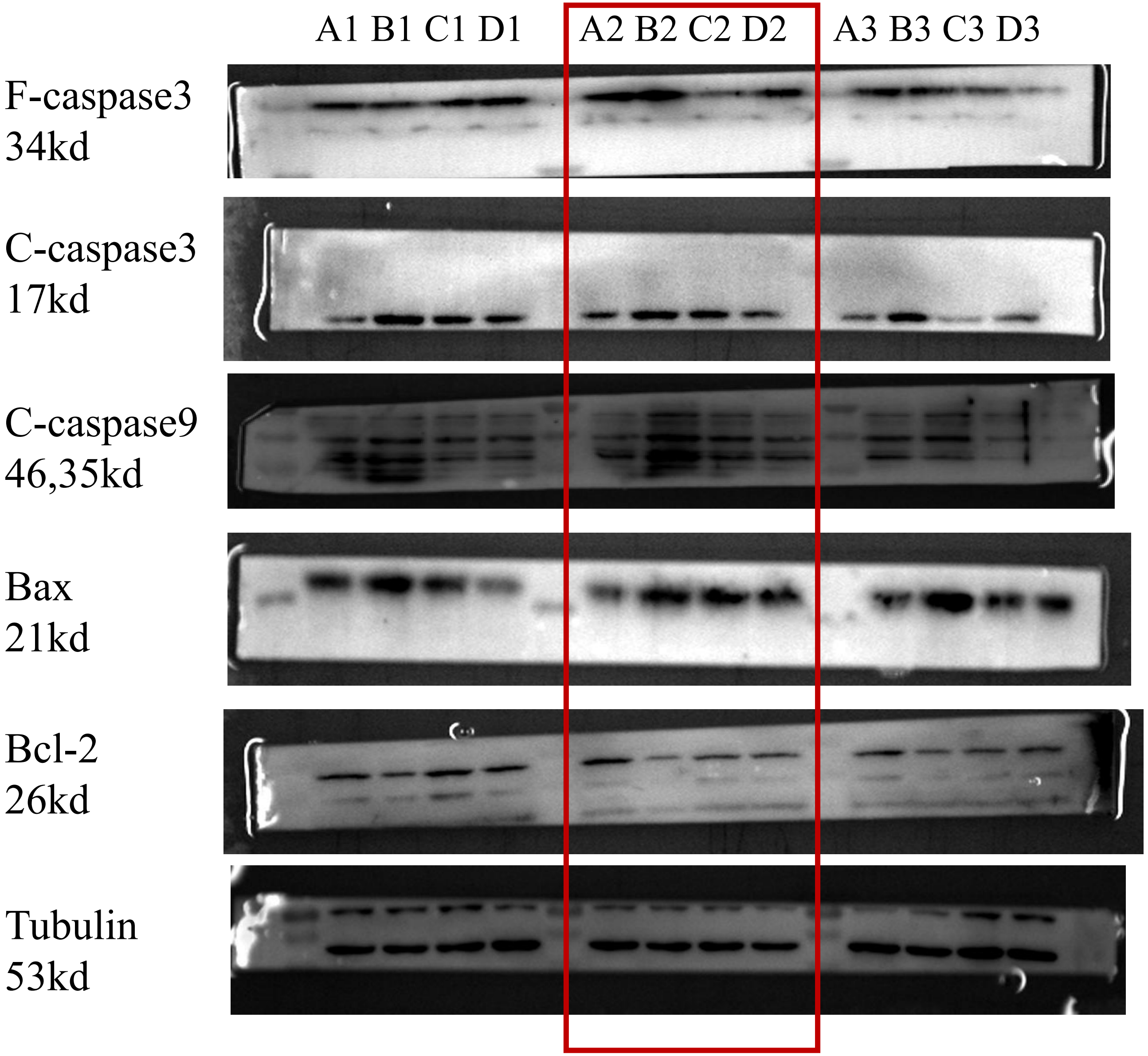

FigureS1(A : Control; B : PrP106-126; C :Irisin+PrP106-126; D : Irisin)

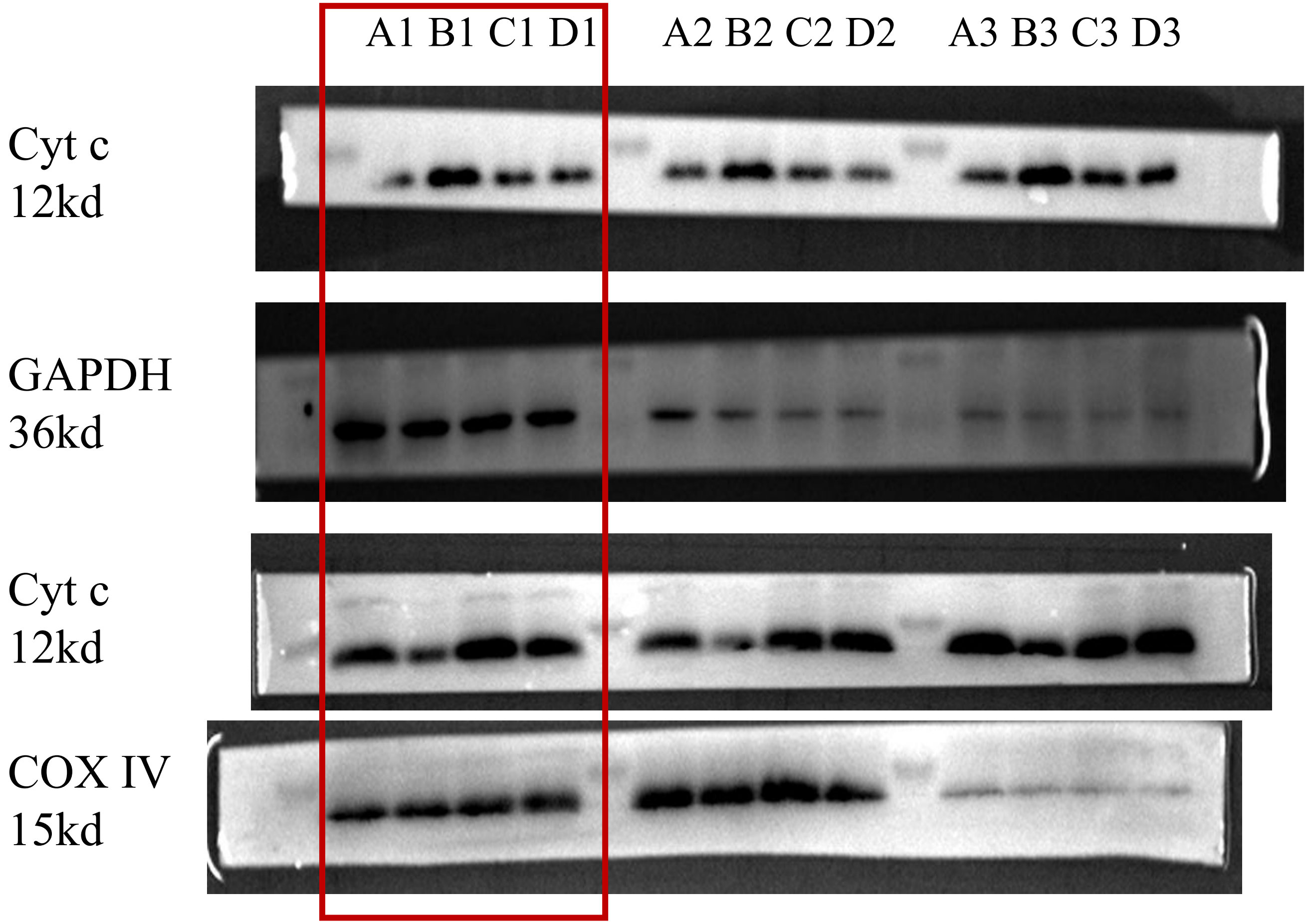

FigureS4C：A-C：Control，D-E：siUCP2

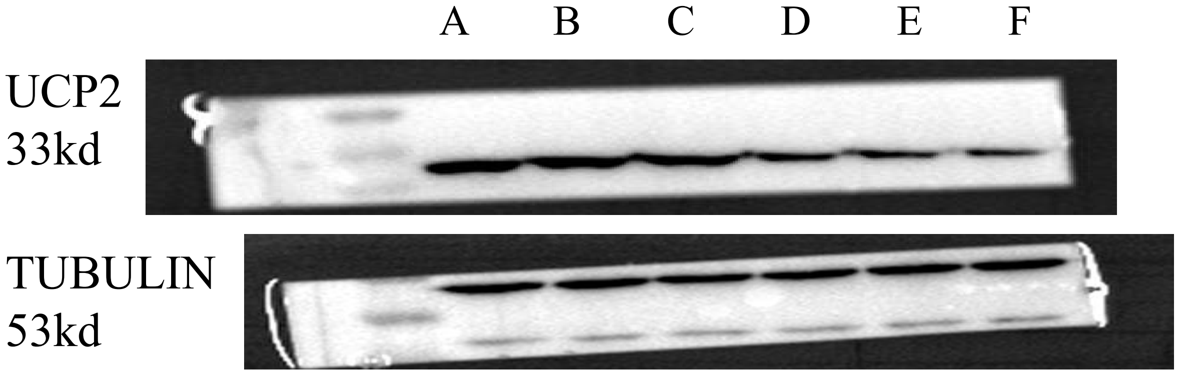

FigureS4C：A-C：Control，D-E：OEUCP2

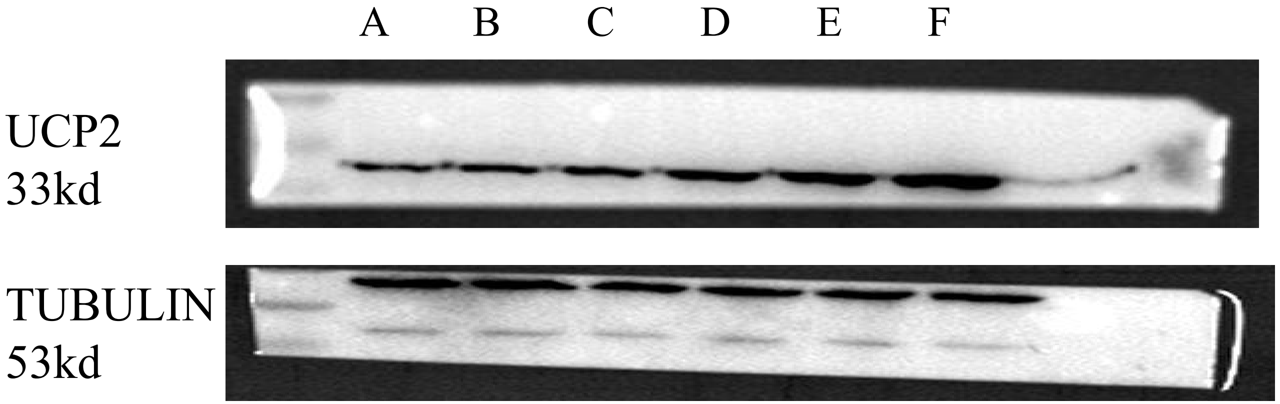

FigureS6A (A : Control; B : PrP106-126; C :Irisin+PrP106-126; D : Irisin)

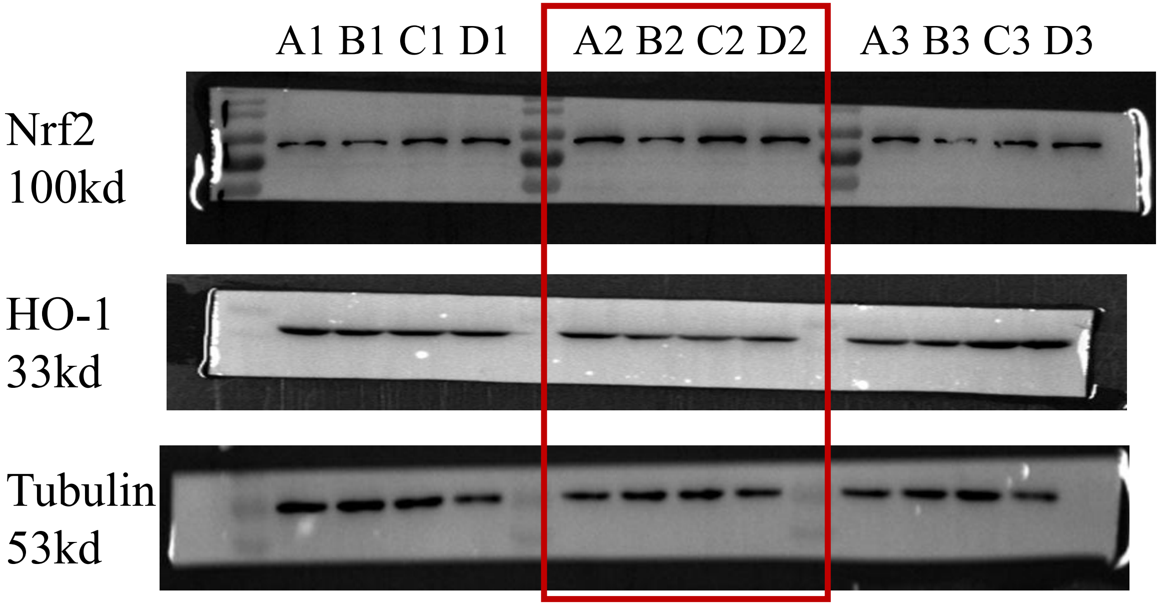

FigureS6C (A : Control; B : PrP106-126; C :Irisin+PrP106-126; D : Irisin)

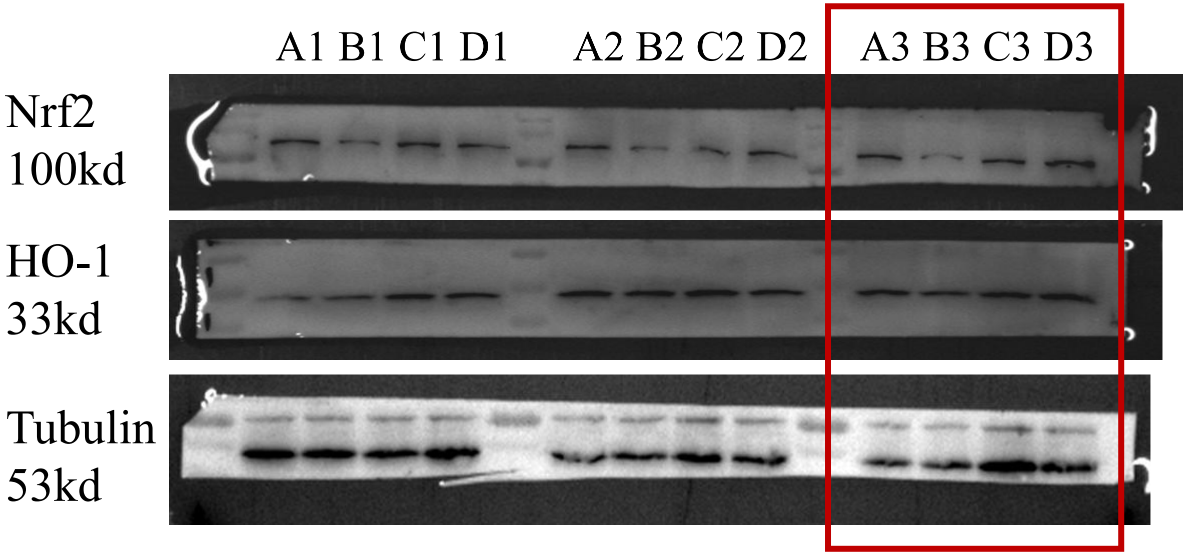

FigureS6E：A-C：Control；D-E:siNRF2

A B C D E F

Nrf2
100kd

Tubulin
53kd

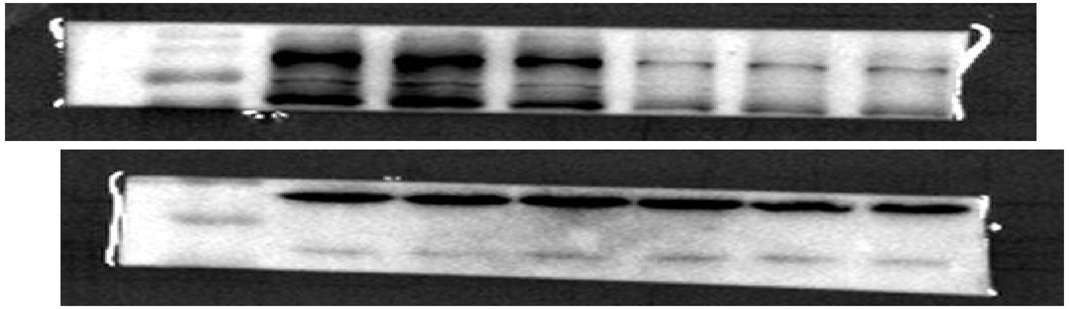

FigureS6E：A-C：Control；D-E：OENRF2

A B C D E F

OENrf2
100kd

Tubulin
53kd

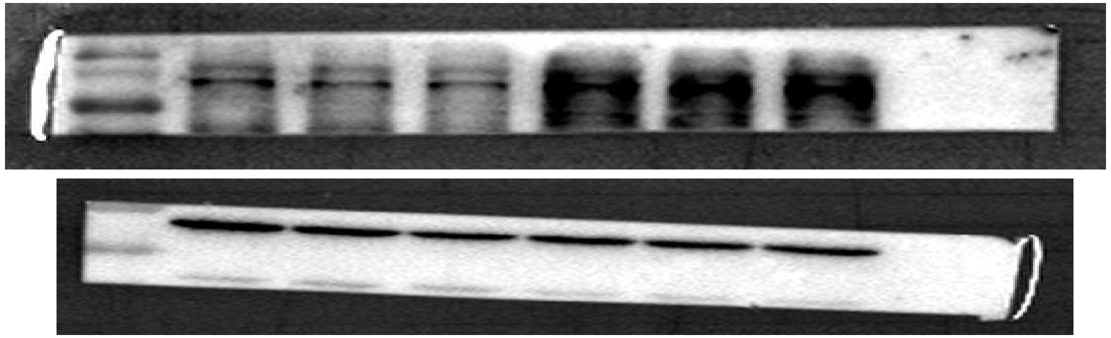

FigureS6F：(A：Control；B：PrP106-126；C：Irisin+PrP106-126；
D：PrP106-126+Irisin+siNRF2; E：PrP106-126+siNRF2：
F：Ctlr siRNA；G：PrP106-126+OENRF2)

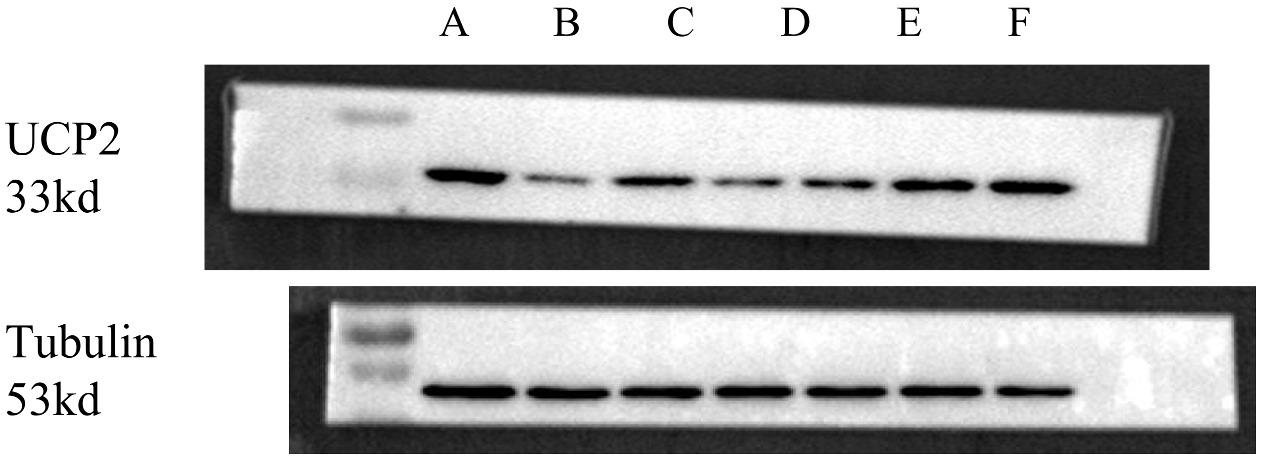

Figure8A (A : Control; B : PrP106-126; C :Irisin+PrP106-126; D : Irisin)

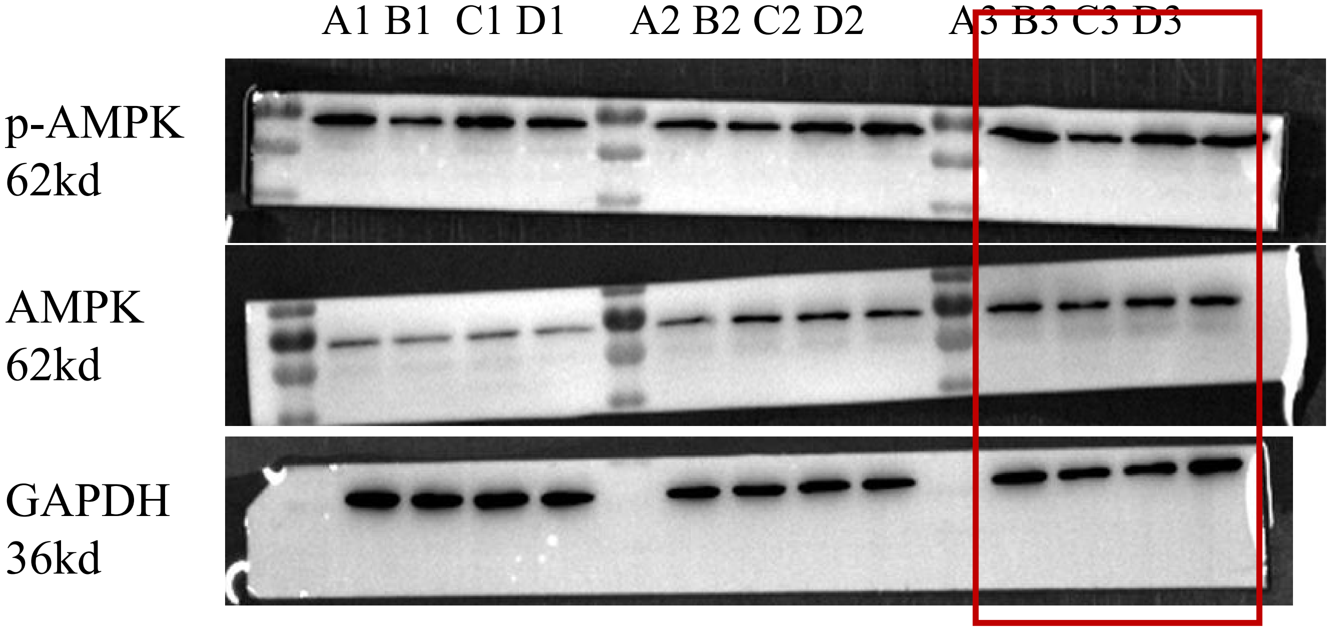

FigureS8B (A : Control; B : PrP106-126; C :Irisin+PrP106-126; D : Irisin)

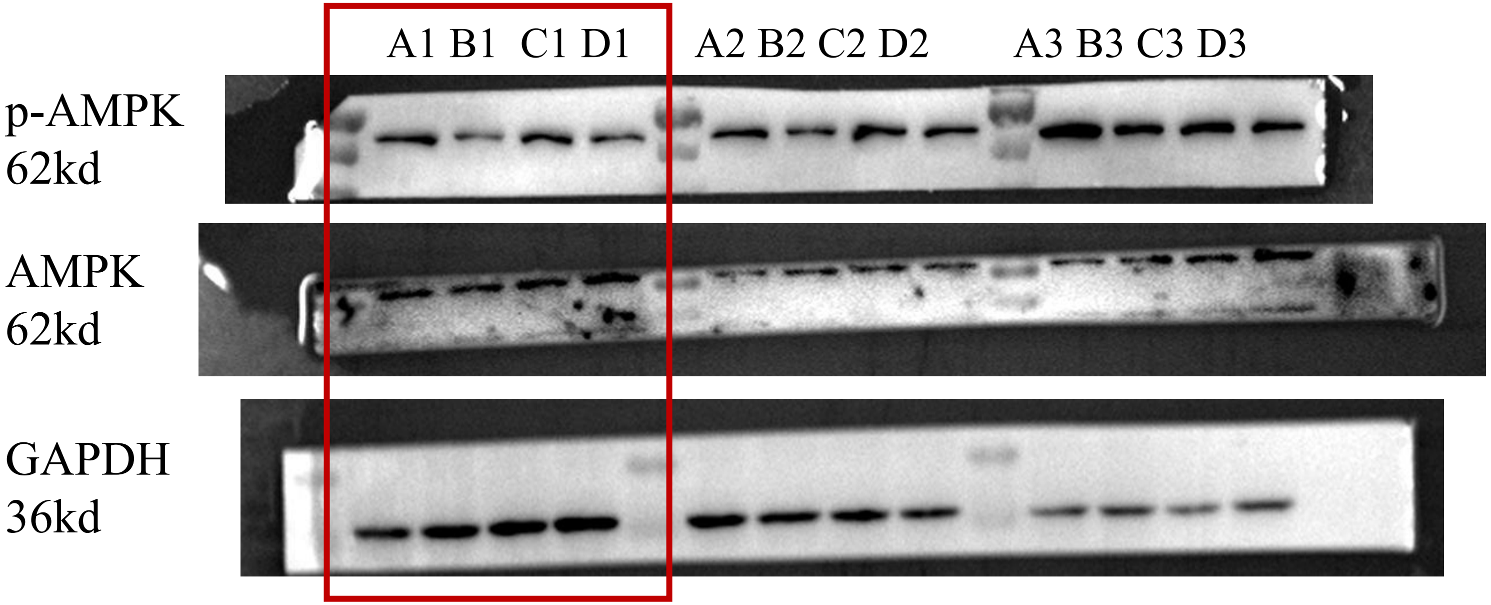

FigureS8C (A : Control; B : PrP106-126; C :Irisin+PrP106-126; D : Irisin)

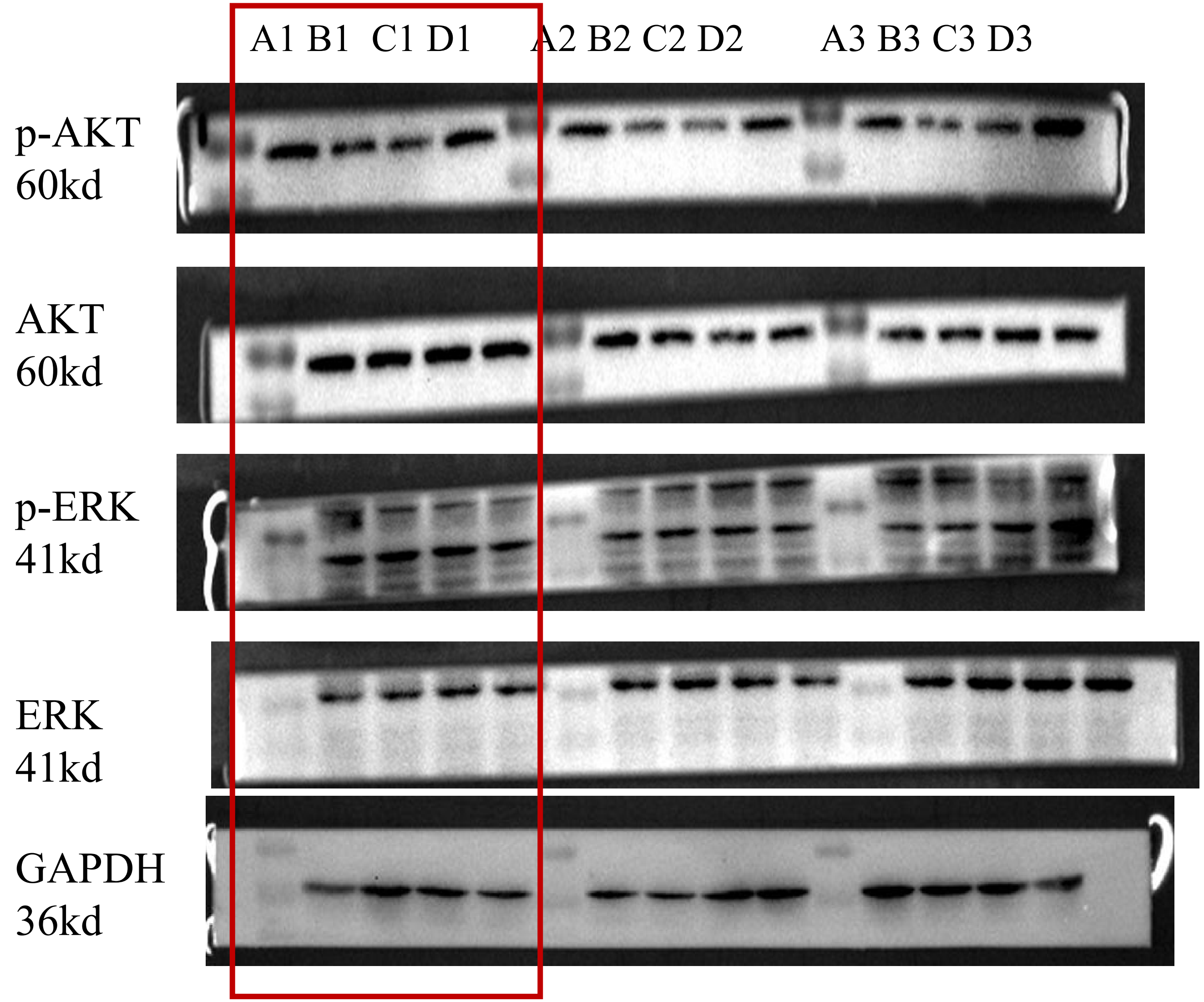

FigureS8D： A-C： Control； D-E： CC

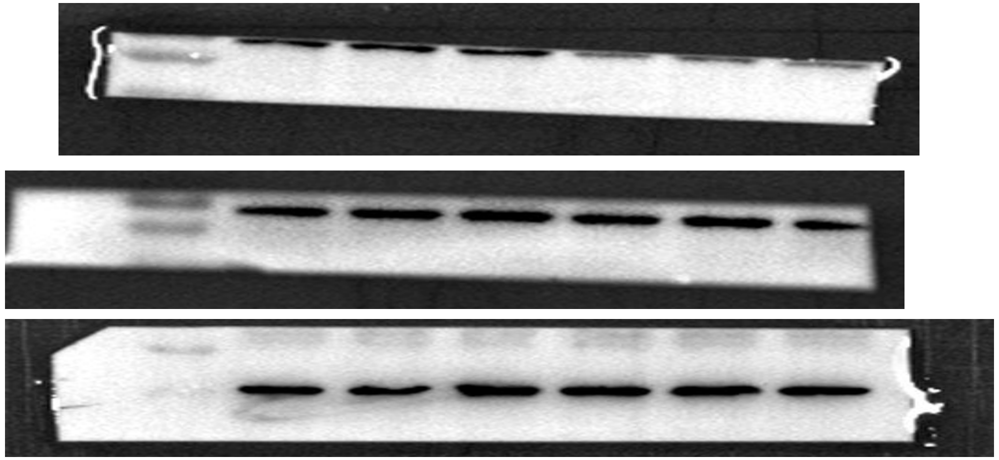

FigureS8D： A-C： Control； D-E： AICAR

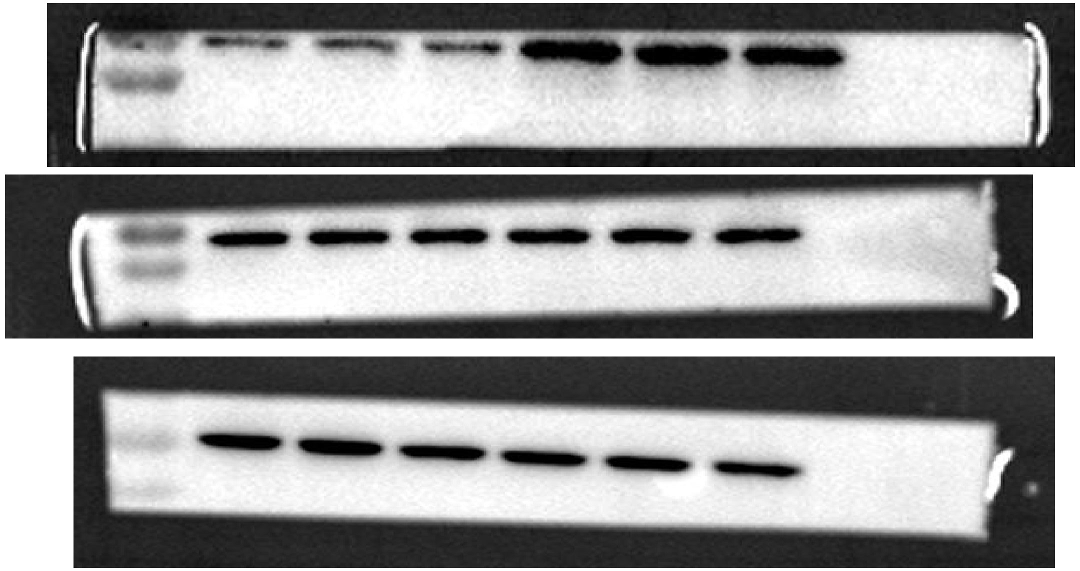

Supplement: Supplementary file 2 — The original Western blot bands [file 41419_2025_7390_MOESM2_ESM.pdf]
